# Application of toasted vine-shoot chips and ultrasound treatment in the ageing of Primitivo wine

**DOI:** 10.1016/j.ultsonch.2024.106826

**Published:** 2024-02-24

**Authors:** Mirella Noviello, Vito Michele Paradiso, Giuseppe Natrella, Giuseppe Gambacorta, Michele Faccia, Francesco Caponio

**Affiliations:** a Department of Soil, Plant and Food Science (DISSPA), University of Bari Aldo Moro, Via Amendola, 165/a, I-70126 Bari, Italy; b Department of Biological and Environmental Sciences and Technologies, University of Salento, S.P. 6, Lecce-Monteroni, I-73100 Lecce, Italy

**Keywords:** Accelerated ageing, Viticultural wastes, Primitivo wine, Sensory analysis

## Abstract

Ageing wine in barrels is an historical practice used to improve the aromatic complexity of wine, but due to the high cost and the long ageing period, alternative approaches have been developed, such as the use of wood chips and ultrasound treatment. The present paper reports the results of an investigation performed on wine (cv. Primitivo). Three treatments were investigated: a) control wine untreated; b) wine with toasted vine-shoot chips (10 g/L); c) wine with toasted vine-shoot chips (10 g/L) and treated by ultrasound. Wines were analysed after 7, 14, 21, and 28 days. The application of ultrasound combined with vine-shoot chips promoted tannin evolution, thereby accelerating the ageing process of wine. The chips addition decreased the total anthocyanins content and increased the stilbenes (*trans*-resveratrol and *trans*-piceid) and wood-related aromas (i.e., furfural, 5-methylfurfural) concentration. Finally, wines added with chips were richer in woody, vanilla, oak, and chocolate notes and more preferred by the tasters.

## Highlights

• Primitivo toasted vine-shoots chips were tested as an alternative to oak chips.
• Primitivo toasted vine-shoots chips and ultrasound treatment were used during wine aging.
• Vine-shoots chips and ultrasound treatment promoted the colour stabilisation, accelerating the aging process of wine.
• Primitivo wine enriched of compounds associated with new wood-related aromas.

## Graphical abstract

**Figure:**
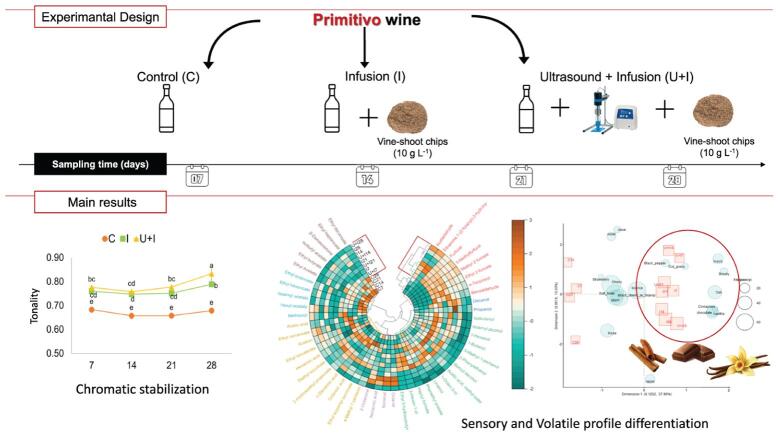

## 1 Introduction

Wine ageing is one of the most valuable phases of winemaking process, essential to produce high-quality red wines. However, the long time and high economic costs of the wine ageing process in barrels has led to the development of several alternatives ageing techniques such as the application of ultrasounds, high pressure, pulsed electric field or the addition of oak products [1].

Ultrasound is one of the most promising technologies in the food sector due to its ample application in different processes, being a low-cost and environmentally friendly technology [2], [3]. In the oenological field, ultrasound has been tested for different purposes, such as: improving the extraction of phenolic and other grape compounds, reducing the processing time (both maceration and ageing) and obtaining wines with a suitable and stable phenolic composition [4], [5], [6], [7], as recently accredited by International Organisation of Vine and Wine (OIV) [8]; reducing the SO_2_ dose in wine production [9]; improving ageing on lees [10], [11], [12], [13]; extracting bioactive compounds from wastes and by-products of the winemaking process [14], [15], [16], [17], [18]. Furthermore, ultrasound treatment has been tested to accelerate the ageing process of wine, improving quality and accelerating colour stabilization and proanthocyanidin polymerization reactions [1], [19], [20], [21]. In fact, ultrasound is a kind of mechanical waves with a frequency above 20 kHz that need a material medium to propagate [22].

On the other hand, the addition of wood chips from *Quercus* species in winemaking was approved and regulated by the International Oenological Codex of the OIV [23] and by the Official Journal of the European Union [24]. Wood chips from other botanical species such as chestnut, acacia or cherry, have also been tested to improve wine quality [25], [26], [27], [28]. Moreover, the use of toasted vine-shoot (*Vitis vinifera* L.) chips, one of the most abundant viticultural wastes from vinification and ageing processes, have been tested during last years in order to improve the phenolic and volatile composition of wine [29], [30], [31], [32]. This application is in an experimental phase. In fact, to the best of our knowledge in Italy the use of vine-shoot chips during the ageing of wine is not allowed by legal regulations.

The combination of ultrasound technology and addition of oak chips has been investigated in model wine solution and during wine ageing processes, resulting in improvement of the aromatic quality, colour, and taste, and increase of the total phenols content [1], [33], [34]. However, the effect of ultrasound process combined with vine-shoot chips during wine ageing has not been studied yet.

Therefore, this work evaluates the effects of the use of toasted vine-shoot chips either combined with ultrasound treatment or not on the ageing phenomena of a Primitivo wine. To the aim, the phenolic and volatile composition, and the sensory profile were monitored weekly over a period of 28 days of contact with vine-shoot chips.

## 2 Materials and methods

### 2.1 Vine-shoot chips preparation

The cv. Primitivo vineyard located in Laterza (Puglia, Italy) (coordinates: longitude 16° 78′ 23.8495″ W, and latitude 40° 69′ 76.1435″ N) was pruned 90 days after grape harvest (season 2021). About 20 kg of vine-shoots were picked randomly and stored intact (40 cm) in dark and room temperature (18 ± 3 °C) condition for six months, to achieve the highest accumulation of non-volatile phenolic and volatile compounds [35]. Then, they were cut into 3–4 cm pieces, ground by a hammer miller (Dietz-motoren KG, Elektromotorenfabrik, 7319 Dettingen-teck, Germany) to a particle size around 2–20 mm long (similar to commonly used oak chips) and finally, toasted (180 °C) using a thermostatic oven (TFC A120 Forced Air Oven, ArgoLab, Carpi, Italy), as described by Cebrián-Tarancón et al. [36]. A slight change was made compared to literature (one hour toasting instead of 45 min), after preliminary trials (data not shown).

### 2.2 Experimental design

A red wine (cv. Primitivo, 20 L) from the 2021 vintage was supplied by the “La Popolare” winery (Sava, Puglia, Italy) and delivered at the experimental winery of the Department of Soil, Plant and Food Sciences (Di.S.S.P.A, University of Bari Aldo Moro, Italy). The general oenological chemical parameters of the wine, before treatment, were: alcoholic degree 15.6 % (v/v), total acidity 6.3 g/L as tartaric acid, pH 3.6, volatile acidity 0.34 g/L as acetic acid, tartaric acid 2.6 g/L, malic acid 0.66 g/L, lactic acid 0.47 g/L, dry reduced extract 38.5 g/L, and ashes 2.7 g/L and 93.9 mg/L of SO_2_.

Three treatments were tested, and twelve 500 mL bottles were sampled for each treatment:

1. C (control): wine without any treatment;
2. I (infusion): wine with 10 g/L of toasted vine-shoot chips. The amount of chips used was lower than that reported by Cebrián-Tarancón et al. [30] (12 g/L) to balance the stronger sensory impact due to the longer toasting time of the chips (see also Section 2.1);
3. U + I (ultrasound + infusion): wine with toasted vine-shoot chips (10 g/L) and subjected to ultrasound treatment for 30 min with a Bandelin Sonopuls GM3200 ultrasound system (Bandelin electronic, Berlin, Germany) at a frequency of 20 kHz and power of 150 W, operating with a 13 mm probe (Bandelin sono plus 497 titanteller TT 13). The temperature was controlled by a thermostatic bath (20 ± 5 °C). The treatment was directly carried out on the wine in the bottles.

For both I and U + I samples, the wine was added together with the chips in each bottle. All bottles (C, I and U + I) were stored at room temperature (20 ± 5 °C) for 28 days with daily shaking. All wines were analysed at 7, 14, 21 and 28 days. Before analysis, I and U + I samples were filtered to remove the chips.

### 2.3 Analytical methods

#### 2.3.1 General oenological parameters

Ethanol (E, % v/v), pH, titratable acidity (TA, g/L tartaric acid), volatile acidity (VA, g/L acetic acid), tartaric acid (TarA, g/L), malic acid (MA, g/L) and lactic acid (LA, g/L), dry reduced extract (DRE, g/L), ashes (g/L), SO_2_ (mg/L) were analysed in triplicate by using a Foss WineScan FT 120, as described by the manufacturer (Foss, Hillerød, Denmark).

#### 2.3.2 Phenolic composition and colour indices

Total phenolic content (TPC as mg/L of gallic acid equivalents) was determined according to Gambacorta et al. [37]. Flavonoids (F, as mg/L of (+)-catechin), anthocyanins (A, as mg/L of malvidin-3-glucoside), flavans reactive with vanillin (FRV, as mg/L of (+)-catechin) and proanthocyanidins (P, as mg/L of cyanidin chloride) were determined according to Gambacorta et al. [38]. Colour indices (T, tonality; CI, colour intensity) were evaluated according to the Glories procedure [39]. An Evolution 60 s UV–visible spectrophotometer (ThermoFisher Scientific, Rodano, Italy) was employed for the spectrophotometric measures.

#### 2.3.3 Antioxidant activity evaluation

Antioxidant activity (AA_DPPH_) was assessed by DPPH (2,2-diphenyl-1-picrylhydrazyl) and ABTS-TEAC assays according to Tarantino et al. [40] using an Evolution 60 s UV–visible spectrophotometer (ThermoFisher Scientific, Rodano, Italy), expressing the results as µmol Trolox equivalents (TE) per L of wines samples.

#### 2.3.4 Analysis of phenolic compounds by UHPLC-DAD-MS/MS

UHPLC-DAD-MS/MS analysis of phenolic compounds was carried out according to Torregiani et al. [41], with the only exception that data were acquired in both positive and negative ionization mode. Specifically, samples were analysed with two methods: a full scan method from 100 to 1000 *m*/*z* and a data-dependent experiment to collect MS^2^ data. In this case the data-dependent settings were full scan from 140 to 800 *m*/*z* for negative ionization mode and from 200 to 1000 for positive ionization., activation level 500 counts, isolation width 2 Da, default charge state 2, and CID energy 35. Tentative identification of compounds was performed using mass spectra (MS^2^), λ_max_, and retention time accordingly to literature [42], [43], [44], [45], [46]. Analytical grade standards were used for quantitation by the external standard method (R^2^ = 0.9972−0.9999): (+)-catechin, (-)-epicatechin, malvidin-3-O-glucoside, quercetin were purchased from phyproof® (PhytoLab, Dutendorfer, Germany); myricetin, gallic acid, caftaric acid, syringic acid, ellagic acid were purchased from Sigma-Aldrich (St. Louis, MO, USA); *trans*-resveratrol was purchased from United States Pharmacopeia (USP, Maryland, United States). Results were expressed in mg of compound per L. All analyses were performed in triplicate.

#### 2.3.5 Volatile organic compounds (VOCs)

VOCs were analysed by SPME-GC/MS according to Prezioso et al [47]. One millilitre of each sample were placed into 20 mL glass vials with a headspace screw cap containing 0.2 g/mL of NaCl (to increase the ionic strength) and 100 µL of internal standard solution (2-octanol, 820 ng) as an internal standard for semi-quantitation and then closed by a silicone/PTFE septum and an aluminium cap. Before extraction, stabilization of the headspace in the vial was obtained by equilibration for 10 min at 50 °C. The extraction was performed using a divinylbenzene/carboxen/polydimethylsiloxane (DVB/CAR/ PDMS) 50/30 mm SPME fiber assembly (Supelco, Bellefonte, PA, USA) at 50 °C for 30 min. The GC–MS analyses were carried out using a Trace1300 gas chromatograph equipped with a mass spectrometer ISQ Series 3.2 SP1. Tentative identification of the peaks was done by means of Xcalibur V2.0 Qual Browse software (Thermo Fisher Scientific, Waltham, MA, USA) by matching with the reference mass spectra of the NIST (National Institute of Standards and Technology, Gaithersburg, MD, USA) library. Semi-quantitation of the compounds was done by the internal standard method, and the amounts were expressed as µg of 2-octanol equivalents per L.

The theoretical influence of the aroma compounds on the overall aroma of wine was evaluated, determining the odour activity value (OAV), as the ratio between the concentration and the odour threshold of the individual aroma compounds found in bibliography, as reported in Table S2 of Supplementary Materials.

#### 2.3.6 Sensory analysis

The effect of the different treatments was evaluated by subjecting the wine samples (C, I and U + I) to sensory analysis. One session was performed for each sampling time considered (7, 14, 21 and 28 days). The evaluation panel was composed of 8 expert tasters (4 women and 4 men) between 24 and 60 years of age, who expressed written consent according to the ethical guidelines of the laboratory of Food Science and Technology of the Department of Soil, Plant, and Food Science of the University of Bari (Italy). In each session, the three wines samples were presented in a completely randomized order to each panellist without giving any information about their preparation. Before the first session, a preliminary consensus session was carried out with a blind simultaneous tasting of three wine samples to highlight relevant differences among wines and define the descriptors to be used in the quantitative descriptive analysis (QDA) and in the list used for CATA analysis. The mean scores of attributes obtained from QDA provided the sensory profile of the wines, according to Iland et al. [48]. For this purpose, an evaluation sheet was provided to judges and the descriptors were grouped by visual (colour intensity and viscosity), olfactory (intensity, persistence, balance) and taste (gustatory intensity, gustatory persistence, tannicity, gustatory balance, and body) characteristics and an overall score. Panellists rated each attribute on a scale from 0 (absence) to 10 (maximum perception). The olfactory profile was also valuated by check-all-that-apply (CATA) approach [49], by which the judges were asked to report the perception of odorous attributes (such as fruity, floral, chocolate, toasty, vanilla, oak). At the end of the session, the assessors were asked to express their preference, creating a ranking of preference from 1 to 3.

### 2.4 Statistical analysis

All results were subjected to two-way analysis of variance (ANOVA) to evaluate the effects of sampling time (Tm) and treatments (Tr) and their interaction (Tm*Tr). A circular polar heatmap was obtained from a hierarchical clustering analysis involving both wines and volatile compounds. The results about the quantitative descriptive analysis were subjected to ANOVA. The results of the preference ranking test were subjected to Friedman analysis. The wines tasted were ranked in descending order of preference for each consumer; rank 1 was assigned to the most preferred wine, rank 2 to the next ones and rank 3 to the least preferred wine. The KH Coder software was used on the results of CATA sensory analysis to achieve the correspondence analysis (minimum term frequency = 3). OriginPro 2020 (OriginLab Corporation, Northampton, MA, USA) was used for the statistical analysis.

## 3 Results and discussion

### 3.1 Oenological parameters

Table 1 shows the effect of sampling time (7, 14, 21, 28 days) and treatments (C, I and U + I). The sampling time statistically affected VA, TarA and DRE, though no clear trend was observed, and, above all, changes had no relevant oenological impact on the parameters. Also, total SO_2_ showed a general tendency to decrease during time, probably due to oxygen solubilization during bottling and treatments, as well as radical formation induced by ultrasound. Treatment exerted a greater effect than sampling time on all parameters, except for malic and lactic acid. Firstly, the ultrasound treatment reduced the alcohol content (*p* < 0.001). The decrease of ethanol was neglectable but provided the evidence of the chemical impact of ultrasound on wine. In fact, it has been reported that the cavitation effect may cause oxidation of ethanol and formation of free radicals which could contribute to the acceleration of wine ageing reactions [50], [51], [52]. Moreover, both treatments favoured a slight increase of pH, DRE and ashes, probably due to the release of solids and cations from chips. Although to differing degrees, the pH increment was observed also when Airén and Cencibel vine-shoots were tested in a model wine solution [29].

**Table 1:** Incidence of sampling time (Tm, 7, 14, 21, 28 days) and treatment (Tr) on general oenological parameters of Primitivo wine.

	**Days**	**E (% v/v)**	**pH**	**TA (g/L)**	**VA (g/L)**	**TarA (g/L)**	**MA (g/L)**	**LA (g/L)**	**DRE (g/L)**	**Ashes (g/L)**	**SO_2_ (mg/L)**
**C**	**7**	^†^15.60 ± 0.02^a^	3.54 ± 0.02^c^	6.24 ± 0.05^ab^	0.31 ± 0.01^abc^	2.59 ± 0.04^cd^	0.70 ± 0.1^a^	0.47 ± 0.02^a^	38.23 ± 0.23^e^	2.77 ± 0.03^f^	83.33 ± 7.74^ab^
	**14**	15.60 ± 0.03^a^	3.54 ± 0.02^abc^	6.23 ± 0.05^ab^	0.33 ± 0.03^abc^	2.70 ± 0.05^abc^	0.67 ± 0.07^a^	0.46 ± 0.04^a^	38.39 ± 0.16^de^	2.80 ± 0.09^ef^	78.57 ± 1.53^ab^
	**21**	15.63 ± 0.01^a^	3.54 ± 0.01^abc^	6.19 ± 0.01^b^	0.31 ± 0.02^bc^	2.72 ± 0.04^ab^	0.72 ± 0.02^a^	0.46 ± 0.05^a^	38.27 ± 0.05^de^	2.86 ± 0.03^cdef^	85.42 ± 5.74^a^
	**28**	15.63 ± 0.01^a^	3.54 ± 0.01^bc^	6.23 ± 0.03^ab^	0.31 ± 0.02^c^	2.76 ± 0.05^a^	0.71 ± 0.02^a^	0.43 ± 0.05^a^	38.28 ± 0.08^de^	2.83 ± 0.06^def^	70.48 ± 3.90^ab^
**I**	**7**	15.63 ± 0.05^a^	3.58 ± 0.01^a^	6.17 ± 0.05^b^	0.33 ± 0.01^abc^	2.66 ± 0.04^abcd^	0.70 ± 0.1^a^	0.47 ± 0.1^a^	38.74 ± 0.17^bcd^	2.96 ± 0.03^abcd^	70.48 ± 10.51^ab^
	**14**	15.59 ± 0.01^a^	3.58 ± 0.01^a^	6.19 ± 0.07^b^	0.33 ± 0.01^abc^	2.64 ± 0.05^abcd^	0.71 ± 0.01^a^	0.49 ± 0.07^a^	38.61 ± 0.20^cde^	3.01 ± 0.06^ab^	77.23 ± 2.43^ab^
	**21**	15.63 ± 0.02^a^	3.58 ± 0.01^ab^	6.26 ± 0.03^ab^	0.35 ± 0.01^ab^	2.62 ± 0.02^bcd^	0.66 ± 0.02^a^	0.47 ± 0.05^a^	39.04 ± 0.03^abc^	2.92 ± 0.04^bcde^	75.66 ± 6.52^ab^
	**28**	15.58 ± 0.02^a^	3.57 ± 0.01^abc^	6.20 ± 0.01^b^	0.31 ± 0.01^abc^	2.67 ± 0.06^abcd^	0.70 ± 0.01^a^	0.49 ± 0.04^a^	38.72 ± 0.20^bcde^	2.99 ± 0.01^abc^	72.95 ± 6.45^ab^
**U + I**	**7**	15.29 ± 0.04^b^	3.57 ± 0.01^abc^	6.27 ± 0.04^ab^	0.33 ± 0.01^abc^	2.57 ± 0.02^d^	0.70 ± 0.02^a^	0.51 ± 0.04^a^	39.20 ± 0.26^ab^	3.03 ± 0.03^a^	80.14 ± 3.40^ab^
	**14**	15.32 ± 0.01^b^	3.58 ± 0.01^ab^	6.18 ± 0.04^b^	0.33 ± 0.01^abc^	2.69 ± 0.03^abc^	0.71 ± 0.06^a^	0.44 ± 0.03^a^	39.04 ± 0.04^abc^	3.07 ± 0.03^a^	75.90 ± 3.84^ab^
	**21**	15.27 ± 0.05^b^	3.58 ± 0.01^ab^	6.26 ± 0.03^ab^	0.35 ± 0.02^a^	2.71 ± 0.02^ab^	0.71 ± 0.05^a^	0.50 ± 0.04^a^	39.40 ± 0.24^a^	3.07 ± 0.04^a^	74.67 ± 4.47^ab^
	**28**	15.30 ± 0.04^b^	3.58 ± 0.01^a^	6.32 ± 0.06^a^	0.33 ± 0.01^abc^	2.64 ± 0.03^bcd^	0.72 ± 0.04^a^	0.49 ± 0.01^a^	39.53 ± 0.17^a^	3.06 ± 0.06^a^	66.74 ± 6.06^b^
	**Tm**	ns	ns	ns	*	***	ns	ns	*	ns	*
*Significance*	**Tr**	***	***	**	**	*	ns	ns	***	***	ns
	Tm*Tr	ns	ns	*	ns	**	ns	ns	*	ns	ns

^†^Different letters in columns indicate statistically significant differences at *p <* 0.05 according to two-way ANOVA with interaction followed by Tukey’s test. Significance: ns, *, **, and ***, not significant or significant at *p* < 0.05, *p* < 0.01, or *p* < 0.001, respectively. Average value ± standard deviation (n = 3). *Abbreviations*: C, control wine; I, wine with infusion of vine-shoot chips; U + I, wine with ultrasound and infusion of vine-shoot chips. E, Ethanol; TA, titratable acidity: as tartaric acid; VA, volatile acidity: as acetic acid; TarA, tartaric acid; MA, malic acid; LA, lactic acid; DRE, dry reduced extract.

Also, TA, VA, TarA slightly changed compared to control, without considerable oenological impact. As regards the interaction between time and treatment (Tm*Tr), significant variations were observed for TA, TarA and DRE, though, also in this case, variations were of low oenological relevance.

### 3.2 Phenolic composition, antioxidant activity and colour indices

The results for phenolic composition, antioxidant activity and colour indices analysed by a two-way ANOVA, considering interactions between sampling time (Tm) and treatments (Tr), are shown in Table 2. Both treatment and time influenced most of the parameters considered. Tm*Tr interactions resulted significant for all phenolic parameters (except for F and FRV), antioxidant activity and colour indices. This result indicated that the effect of the vine-shoot treatments applied depended upon the contact time with chips. The evolution of all these parameters has been also represented in Fig. S1 of Supplementary Materials.

**Table 2:** Incidence of sampling time (Tm, 7, 14, 21, 28 days) and treatments (Tr) on phenolic composition, activity antioxidant and colour indices of Primitivo wines.

	**Days**	**TPC** **(mg/L)**	**A** **(mg/L)**	**F** **(mg/L)**	**AA_DPPH_** **(µmol trolox eq/L)**	**AA_ABTS_** **(µmol trolox eq/L)**	**FRV** **(mg/L)**	**P** **(mg/L)**	**FRV/P**	**CI**	**T**
**C**	**7**	^†^2821.8 ± 116.0^abc^	346.0 ± 6.9^a^	2600.5 ± 33.5^ab^	11850.0 ± 325.0^d^	17159.5 ± 288.7^cd^	1605.7 ± 10.3^abc^	2746.1 ± 54.0^abc^	0.58 ± 0.01^a^	0.99 ± 0.01^a^	0.68 ± 0.01^e^
	**14**	2863.1 ± 116.6^ab^	333.7 ± 2.5^ab^	2488.4 ± 9.1^abcd^	14366.7 ± 312.6^bc^	17094.0 ± 226.1^cd^	1613.9 ± 77.1^ab^	2877.1 ± 56.5^abc^	0.56 ± 0.03^ab^	0.91 ± 0.01^de^	0.66 ± 0.01^e^
	**21**	2769.4 ± 50.1^abcd^	336.8 ± 11.0^ab^	2670.0 ± 94.1^a^	14833.3 ± 570.3^bc^	18594.0 ± 116.2^a^	1517.0 ± 20.4^bcd^	3041.5 ± 143.5^a^	0.50 ± 0.02 ^cd^	0.94 ± 0.01^cd^	0.66 ± 0.01^e^
	**28**	2756.3 ± 6.6^abcd^	311.7 ± 8.7^bc^	2503.5 ± 33.9^abcd^	15525.0 ± 597.4^ab^	18385.7 ± 71.4^ab^	1690.0 ± 14.6^a^	2920.4 ± 137.1^ab^	0.58 ± 0.02^ab^	0.98 ± 0.01^ab^	0.68 ± 0.01^e^
**I**	**7**	2964.3 ± 109.9^a^	297.4 ± 8.7^cd^	2560.2 ± 46.7^abc^	16250.0 ± 350.0^a^	15403.6 ± 146.2^e^	1409.4 ± 32.7^de^	2679.3 ± 35.2^cde^	0.53 ± 0.01^bc^	0.95 ± 0.01^bc^	0.76 ± 0.01^cd^
	**14**	2573.1 ± 37.9 ^cd^	259.7 ± 3.3^e^	2405.4 ± 12.3^cde^	13691.7 ± 170.2^c^	17207.1 ± 236.2^cd^	1442.9 ± 19.7^de^	2425.4 ± 59.1^f^	0.60 ± 0.02^a^	0.81 ± 0.01^h^	0.75 ± 0.01^d^
	**21**	2592.4 ± 30.2^bcd^	267.1 ± 9.5^e^	2470.2 ± 91.0^bcd^	14141.7 ± 464.6^c^	17689.3 ± 357.1^bc^	1334.3 ± 37.4^e^	2530.1 ± 22.9^def^	0.53 ± 0.01^bc^	0.83 ± 0.01^gh^	0.75 ± 0.01^cd^
	**28**	2578.0 ± 100.2^bcd^	230.6 ± 2.3^f^	2323.8 ± 27.8^de^	14200.0 ± 625^c^	17397.6 ± 168.8^cd^	1454.5 ± 45.6^cde^	2461.1 ± 26.0^ef^	0.59 ± 0.01^a^	0.87 ± 0.01^fg^	0.79 ± 0.01^b^
**U + I**	**7**	2768.0 ± 219.1^abcd^	277.5 ± 8.0^de^	2446.3 ± 100.0^bcde^	14975.0 ± 442.3^abc^	15698.2 ± 116.1^e^	1373.1 ± 31.6^de^	2402.3 ± 46.1^f^	0.57 ± 0.01^ab^	0.96 ± 0.01^abc^	0.78 ± 0.01^bc^
	**14**	2693.0 ± 88.8^abcd^	258.7 ± 6.7^e^	2419.1 ± 70.5^bcde^	13858.3 ± 610.5^c^	16885.7 ± 523.4^d^	1426.4 ± 16.0^de^	2439.8 ± 91.6^f^	0.59 ± 0.03^a^	0.82 ± 0.01^h^	0.76 ± 0.01^cd^
	**21**	2557.3 ± 10.4^cd^	255.8 ± 12.6^e^	2491.2 ± 86.6^abcd^	13875.0 ± 132.3^c^	17415.5 ± 149.8^cd^	1335.3 ± 6.9^e^	2807.7 ± 7.7^abc^	0.48 ± 0.01^cd^	0.88 ± 0.01^ef^	0.78 ± 0.01^bc^
	**28**	2494.6 ± 52.0^d^	212.0 ± 13.4^f^	2272.4 ± 34.5^e^	14425 ± 278.4^bc^	16885.7 ± 280.6^d^	1375.0 ± 53.6^de^	3037.7 ± 128.1^a^	0.45 ± 0.03^d^	0.89 ± 0.01^ef^	0.83 ± 0.03^a^
	**Tm**	***	***	***	*	***	***	***	***	***	***
*Significance*	**Tr**	***	***	***	ns	***	***	***	***	***	***
	Tm*Tr	*	*	ns	***	***	ns	***	***	***	***

^†^Different letters in columns indicate statistically significant differences at *p* < 0.05 according to two-way ANOVA with interaction followed by Tukey’s test. Significance: ns, *, **, and ***, not significant or significant at *p* < 0.05, *p* < 0.01, or *p* < 0.001, respectively. Average value ± standard deviation (n = 3). *Abbreviations*: C, control wine; I, wine with infusion of vine-shoot chips; U + I, wine with ultrasound and infusion of vine-shoot chips; TPC, total phenolic content: as gallic acid equivalents; A, anthocyanins: as malvidin-3-glucoside; F, flavonoids: as (+)-catechin; antioxidant activity by DPPH and ABTS assay; FRV, flavans reactive with vanillin: as (+)-catechin; P, proanthocyanidins: as cyanidin chloride; CI, colour intensity; T, tonality.

In general, the addition of vine-shoot chips caused the reduction of total phenolic content (TPC), anthocyanins (A), flavonoids (F), antioxidant activity (ABTS assay), flavans reactive with vanillin (FRV), proanthocyanidins (P) and colour intensity (CI). In particular, the TPC of I wine, decreased after 28 days of contact with chips, compared to 7 days of contact, with a similar value than wine U + I. The reduction of phenolic fraction could be correlated to the adsorption/precipitation of these compounds, mainly anthocyanins, onto the chips, as reported in previous studies [28], [32]. Although to a lesser extent, wine C also suffered a decrease in TPC and A during the storage time. This downtrend of phenolic fraction during time could be due to processes of polymerisation, oxidation, and complexation occurring in wine [53]. However, C wine showed higher values of A during storage, compared to the other samples treated with chips. The A decrease was more marked in wines treated with ultrasound. Closely related to A were colour indices. Both treatments resulted in a reduction in CI (*p* < 0.001) and an increase in tonality (T) (*p* < 0.001). The CI decreased in the wines I and U + I during the time of contact with chips, indicating a more rapid evolution of colour. Moreover, the contact with vine-shoot chips determined higher levels of T than the control, which increased over time, especially for U + I wine. In particular, wine U + I presented the highest T, followed by I and C. T is indicative of the colour evolution of red wine according to the prevalence of yellow tones (420 nm) on red tones (520 nm) [54]. In general, the increase of T value indicated the shift to orange tone, which determine the brick-red colour typical of aged wines [55]. The increase of T in wine added with toasted vine-shoot chips was in agreement with previous studies in which red wines were aged with chips of different wood origins [28], [32], [56]. These changes in colour characteristics can be linked to the reduction in the content of free anthocyanins responsible for the red colour, and to several extractable wood components able to react with anthocyanins to form more stable polymeric complexes contributing to the improvement and stabilization of colour [28], [57], [58]. Moreover, it is worth emphasizing that the colour characteristics and anthocyanin concentration were both particularly affected by ultrasound treatment. This could be attributed to a greater extraction of wood molecules which react with anthocyanins, as reported before, and to degradation and polymerization reactions induced by ultrasound [52]. As regards P, their content in the U + I wine increased during contact time: P had the lowest value at 7 days and the highest at 28 days compared to the other two treatments. The effect of contact with vine-shoot chips on FRV/P ratio differed in the treatments under comparison: C wine showed the highest level after 7 days and at 28 days, comparable to I wine. On the other hand, U + I wine was characterized by the lowest value after 28 days, that is indicative of a wine with less astringency. These results agree with those reported by Gambacorta et al. [4], where the sonication process reduced the FRV/P ratio and increased the proanthocyanidins content. It is well known that a low FRV/P ratio characterizes wines suitable of chromatic and tannic stabilization [59]. Therefore, ultrasonication could accelerate the ageing process of wine. Considering DPPH assay the antioxidant activity increased after 28 days for C wine, which showed the highest value of this parameters compared to the treated wines. Otherwise in all wine the antioxidant activity increased after 28 days when considering the ABTS assay. This difference could be explained by the different phenolic composition of the wines over time. Indeed, although the two assays are very similar, the antioxidant activity of certain classes of phenolic compounds (i.e., dihydroalcohols and flavonones) can be better determined by ABTS than DPPH assay [60].

### 3.3 Phenolic profile

Table 3 shows the composition of phenolic compounds of wines, which were grouped in different chemical groups: flavonoids (anthocyanins, flavonols, flavanols), phenolic acids and stilbenes. In general, the prolonged contact between wine and vine shoots caused a decrease in the phenolic fraction. This result agrees with the research of Cebrián-Tarancón et al. [31], in which the addition of vine-shoot chips during winemaking generated lower levels of total low molecular weight phenolic compounds than those of the control wines.

**Table 3:** Incidence of sampling time (Tm, 7, 14, 21, 28 days) and treatments (Tr) on phenolic profile of Primitivo wines.

	**C**				**I**				**U + I**				**Tm**	**Tr**	Tm*Tr
**Anthocyanins (mg/L)**	**7**	**14**	**21**	**28**	**7**	**14**	**21**	**28**	**7**	**14**	**21**	**28**			
De-3-glc^2^	^†^14.9 ± 1.7^ab^	16.8 ± 0.7^a^	13.1 ± 2.5^bcd^	13.2 ± 0.5^bcd^	14.2 ± 0.9^abc^	12.2 ± 0.1^bcde^	10.3 ± 0.2^defg^	8.0 ± 1.5^g^	13.2 ± 0.04^bcd^	11.7 ± 0.6^cdef^	9.8 ± 0.08^efg^	8.6 ± 0.3 ^fg^	***	***	*
Pet-3-glc^2^	35.8 ± 5.2^ab^	38.4 ± 1.1^a^	31.2 ± 5.8^abcd^	30.2 ± 1.7^bcde^	34.1 ± 0.2^abc^	27.6 ± 0.5^cdef^	24.3 ± 0.1^defg^	19.2 ± 3.2^g^	31.2 ± 0.2^abcd^	28.6 ± 0.4^bcde^	23.2 ± 0.6^efg^	20.2 ± 0.1 ^fg^	***	***	ns
Peo-3-glc^2^	20.5 ± 3.0^abc^	21.5 ± 1.2^ab^	18.6 ± 1.4^abcd^	19.8 ± 0.4^abc^	22.3 ± 0.2^a^	16.6 ± 0.6^cde^	14.3 ± 0.2^def^	11.6 ± 3.3^f^	17.8 ± 0.2^abcd^	16.0 ± 0.3^cdef^	12.8 ± 0.2^ef^	17.3 ± 2.9^bcde^	***	***	***
Mav-3-glc^1^	459.5 ± 37.4^cd^	565.1 ± 12.9^a^	509.7 ± 14.2^b^	447.2 ± 19.6^cd^	512.3 ± 9.5^b^	453.0 ± 3.1^cd^	378.1 ± 1.7^e^	339.6 ± 8.8^ef^	483.7 ± 2.7^bc^	438.2 ± 5.3^d^	368.5 ± 5.4^e^	320.9 ± 4.9^f^	***	***	***
VitA^2^	14.9 ± 2.6^ab^	16.1 ± 0.5^a^	15.1 ± 2.7^a^	15.1 ± 0.6^a^	15.0 ± 0.2^a^	13.7 ± 0.3^ab^	13.9 ± 0.2^ab^	11.1 ± 2.2^b^	13.9 ± 0.5^ab^	14.4 ± 0.5^ab^	13.6 ± 0.4^ab^	13.3 ± 0.3^ab^	*	**	*
VitB^2^	5.7 ± 1.2^ab^	5.5 ± 0.3^ab^	4.2 ± 1.0^bcd^	6.0 ± 0.1^a^	5.3 ± 0.2^abc^	4.5 ± 0.03^bcd^	5.3 ± 0.3^ab^	3.6 ± 0.4^d^	4.7 ± 0.2^abcd^	4.6 ± 0.3^abcd^	3.8 ± 0.1 ^cd^	4.6 ± 0.1^abcd^	*	***	***
Mv-3-O-glc-(epi)cat^2^	9.4 ± 1.2^ab^	5.8 ± 0.3^d^	4.7 ± 1.1d^ef^	7.5 ± 0.3^c^	9.8 ± 0.1^a^	5.0 ± 0.1^de^	8.2 ± 0.2^bc^	3.5 ± 0.6^f^	5.5 ± 0.2^d^	4.9 ± 0.1^def^	3.9 ± 0.1^ef^	3.9 ± 0.01^ef^	***	***	***
Pet-3-(6′'-act)-glc^2^	3.9 ± 0.7^a^	3.9 ± 0.3^a^	3.2 ± 0.9^ab^	3.6 ± 0.1^ab^	4.1 ± 0.02^a^	3.3 ± 0.1^ab^	3.5 ± 0.05^ab^	2.5 ± 0.5^b^	3.5 ± 0.2^ab^	3.3 ± 0.1^ab^	2.3 ± 0.9^b^	3.1 ± 0.02^ab^	**	*	*
Peo-3-(6′'-act)-glc^2^	3.5 ± 0.4^ab^	3.5 ± 0.1^ab^	3.0 ± 0.7^abc^	3.3 ± 0.03^abc^	3.6 ± 0.03^a^	2.6 ± 0.1^cde^	3.2 ± 0.02^abc^	1.9 ± 0.4^de^	3.0 ± 0.03^abc^	2.7 ± 0.2^bcd^	2.1 ± 0.01^de^	1.8 ± 0.1^e^	***	***	***
Mv-3-(6′'-act)-glc^2^	53.7 ± 7.4^ab^	57.7 ± 2.0^a^	48.1 ± 10.0^abcd^	48.3 ± 0.7^abcd^	52.4 ± 0.6^abc^	45.1 ± 0.9^bcde^	38.3 ± 0.1^def^	20.6 ± 3.1^g^	47.5 ± 0.7^abcd^	41.7 ± 0.6^cdef^	34.8 ± 0.4^ef^	31.0 ± 0.03 ^fg^	***	***	***
Pet-3-(6′'-t-cm)-glc^2^	9.0 ± 0.4^b^	10.3 ± 0.3^a^	9.0 ± 0.2^b^	9.2 ± 0.3^b^	7.6 ± 0.1^c^	5.9 ± 0.1^de^	5.4 ± 0.2^e^	2.4 ± 0.3^g^	6.5 ± 0.3^d^	5.7 ± 0.2^e^	3.5 ± 0.2^f^	4.1 ± 0.02^f^	***	***	***
Mv-3-(6′'-c-cm)-glc^2^	2.9 ± 0.2^bc^	3.5 ± 0.1^ab^	2.8 ± 0.7^c^	3.7 ± 0.1^a^	3.6 ± 0.02^ab^	2.5 ± 0.05^cd^	2.7 ± 0.02^cd^	1.5 ± 0.03^e^	2.4 ± 0.1^cd^	2.5 ± 0.1 ^cd^	2.0 ± 0.2^de^	2.1 ± 0.1^de^	***	***	***
Peo-3-(6′'-t-cm)-glc^2^	7.5 ± 0.5^ab^	8.3 ± 0.7^a^	5.9 ± 2.2^bc^	6.9 ± 0.2^ab^	5.7 ± 0.2^bc^	4.2 ± 0.1^cd^	2.4 ± 0.01^def^	1.7 ± 0.2^f^	4.7 ± 0.2^c^	3.9 ± 0.1^cde^	1.9 ± 0.1^ef^	2.6 ± 0.3^def^	***	***	*
Mv-3-(6′'-t-cm)-glc^2^	55.8 ± 3.5^ab^	62.9 ± 2.7^a^	46.5 ± 14.9^bc^	47.7 ± 1.4^bc^	43.2 ± 0.1^bc^	37.3 ± 0.7^c^	23.1 ± 0.3^de^	14.5 ± 1.8^e^	40.1 ± 2.0^c^	36.0 ± 0.1^cd^	20.1 ± 1.0^e^	20.0 ± 0.4^e^	***	***	*
***Total***	**696.7 ± 36.6^bc^**	**819.9 ± 20.3^a^**	**710.4 ± 63.2^b^**	**661.7 ± 24.3^bcd^**	**733.1 ± 10.1^b^**	**633.4 ± 3.7^cd^**	**543.1 ± 17.9^ef^**	**441.9 ± 15.3^g^**	**677.8 ± 5.0^bcd^**	**614.3 ± 8.4^de^**	**502.5 ± 6.3 ^fg^**	**454.4 ± 8.0 ^g^**	***	***	***
**Phenolic acids (mg/L)**															
Gallic acid^1^	228.5 ± 36.2^abc^	247.1 ± 5.9^ab^	241.0 ± 9.5^abc^	224.2 ± 7.5^bc^	242.2 ± 1.0^abc^	252.7 ± 2.6^ab^	217.7 ± 7.0^bc^	206.3 ± 21.8^c^	237.9 ± 9.4^abc^	255.4 ± 3.3^ab^	216.0 ± 1.1^bc^	266.6 ± 8.2^a^	**	*	***
Caftaric acid^1^	102.3 ± 12.1^ab^	108.1 ± 3.6^a^	85.6 ± 17.3^b^	94.3 ± 3.5^ab^	104.4 ± 3.3^ab^	106.8 ± 1.1^a^	95.3 ± 3.0^ab^	84.3 ± 8.6^b^	102.8 ± 2.3^ab^	107.4 ± 1.8^a^	96.7 ± 1.1^ab^	113.4 ± 5.7^a^	***	*	*
*cis*-Coutaric acid^3^	14.6 ± 2.2^a^	15.8 ± 0.03^a^	15.3 ± 3.3^a^	14.3 ± 0.3^a^	15.0 ± 0.6^a^	15.7 ± 0.5^a^	14.4 ± 0.3^a^	14.5 ± 0.4^a^	14.0 ± 1.8^a^	15.9 ± 0.1^a^	16.2 ± 0.9^a^	16.0 ± 0.01^a^	ns	ns	ns
*cis*-Fertaric acid^3^	10.8 ± 2.3^a^	11.7 ± 0.3^a^	13.0 ± 3.6^a^	11.8 ± 0.4^a^	12.2 ± 0.2^a^	11.2 ± 0.2^a^	11.0 ± 0.3^a^	10.4 ± 0.3^a^	12.0 ± 0.04^a^	11.6 ± 0.2^a^	11.4 ± 0.1^a^	11.8 ± 0.1^a^	ns	ns	ns
*trans*-Fertaric acid^3^	5.5 ± 0.5^ab^	6.2 ± 0.2^ab^	7.1 ± 2.8^a^	5.5 ± 0.1^ab^	5.9 ± 0.2^ab^	5.2 ± 0.1^ab^	4.9 ± 0.2^ab^	3.8 ± 0.05^b^	5.4 ± 0.2^ab^	6.0 ± 0.1^ab^	4.5 ± 0.3^b^	6.2 ± 0.05^ab^	ns	*	*
Syringic acid^1^	11.1 ± 0.7^def^	13.6 ± 0.5^a^	10.0 ± 0.2^g^	11.9 ± 0.5^cde^	11.5 ± 0.2^cdef^	13.1 ± 0.2^ab^	12.1 ± 0.1^bc^	11.0 ± 0.2^efg^	11.1 ± 0.1^cdef^	12.0 ± 0.3^cd^	10.6 ± 0.4^fg^	11.3 ± 0.02^cdef^	***	***	***
Ellagic acid^1^	4.8 ± 0.8^cde^	7.0 ± 0.3^ab^	6.7 ± 1.1^abc^	5.5 ± 0.4^bcde^	6.6 ± 0.2^abc^	5.4 ± 0.2^bcde^	4.3 ± 1.5^de^	6.0 ± 0.2^bcd^	5.1 ± 0.5^bcde^	8.5 ± 1.1^a^	5.7 ± 0.6^bcd^	3.5 ± 0.02^e^	***	ns	***
***Total***	**377.5 ± 50.5^abc^**	**409.6 ± 9.2^ab^**	**379.7 ± 21.1^abc^**	**367.5 ± 10.8^bc^**	**398.7 ± 4.7^ab^**	**410.1 ± 1.3^ab^**	**359.9 ± 11.2^bc^**	**336.2 ± 31.5^c^**	**389.3 ± 13.7^abc^**	**416.8 ± 3.7^ab^**	**361.1 ± 1.1^bc^**	**429.9 ± 13.8^a^**	***	*	**
**Flavonols (mg/L)**															
Q3OG^4^	4.1 ± 0.7^ab^	4.3 ± 0.1^a^	3.6 ± 0.5^abc^	3.2 ± 0.4^bcd^	3.6 ± 0.1^abc^	3.3 ± 0.02^abc^	2.1 ± 0.1^d^	2.8 ± 0.6^cd^	4.2 ± 0.6^ab^	3.3 ± 0.03^abc^	2.4 ± 0.3^cd^	3.2 ± 0.04^abc^	***	***	*
Myricetin^1^	4.5 ± 0.4^b^	5.4 ± 0.2^a^	1.6 ± 0.2^d^	1.1 ± 0.1^e^	2.0 ± 0.2^cd^	1.9 ± 0.1 ^cd^	nd	nd	2.3 ± 0.05^c^	1.9 ± 0.1^cd^	nd	nd	***	***	***
Quercetin^1^	3.3 ± 0.3^b^	4.1 ± 0.1^a^	1.4 ± 0.1^cd^	1.3 ± 0.1^cd^	1.5 ± 0.1^c^	1.1 ± 0.1^d^	nd	nd	1.4 ± 0.2^cd^	1.2 ± 0.01^cd^	nd	nd	***	***	***
***Total***	**11.9 ± 1.4^b^**	**13.9 ± 0.2^a^**	**6.6 ± 0.5^cd^**	**5.6 ± 0.1^d^**	**7.0 ± 0.2 ^cd^**	**6.3 ± 0.1^cd^**	**2.1 ± 0.1^e^**	**2.8 ± 0.6^e^**	**7.9 ± 0.8^c^**	**6.4 ± 0.1^cd^**	**2.4 ± 0.3^e^**	**3.2 ± 0.1^e^**	***	***	***
**Flavanols (mg/L)**															
Procyanidin B1^5^	95.8 ± 9.7^ab^	91.5 ± 3.7^ab^	80.7 ± 20.9^bc^	88.4 ± 4.4^abc^	108.0 ± 13.3^a^	87.6 ± 2.3^abc^	82.9 ± 1.9^abc^	75.9 ± 1.1^bc^	101.0 ± 13.4^ab^	87.0 ± 0.9^abc^	76.5 ± 3.3^bc^	64.9 ± 1.1^c^	***	ns	ns
(+) catechin^1^	47.5 ± 4.1^b^	45.5 ± 6.2^e^	51.8 ± 2.7^ab^	44.8 ± 1.8^bc^	37.9 ± 2.4^cd^	57.6 ± 1.7^a^	36.9 ± 0.6^cd^	48.2 ± 4.7^ab^	48.7 ± 7.2^ab^	48.6 ± 2.0^ab^	31.4 ± 1.0^de^	45.8 ± 2.7^bc^	**	*	***
Procyanidin B2^5^	69.1 ± 7.4^b^	68.1 ± 7.0^b^	39.4 ± 7.8^c^	45.9 ± 2.0^c^	83.9 ± 7.0^ab^	71.7 ± 6.8^ab^	46.7 ± 1.2^c^	44.9 ± 1.4^c^	88.1 ± 5.6^a^	68.3 ± 9.1^b^	46.3 ± 1.5^c^	69.1 ± 5.4^b^	***	***	**
	**C**				**I**				**U + I**				**Tm**	**Tr**	Tm*Tr
	**7**	**14**	**21**	**28**	**7**	**14**	**21**	**28**	**7**	**14**	**21**	**28**			
Procyanidin B3^5^	47.5 ± 3.9^a^	51.2 ± 2.3^a^	57.5 ± 2.4^a^	54.0 ± 3.2^a^	52.4 ± 3.1^a^	57.8 ± 6.5^a^	47.4 ± 4.4^a^	60.7 ± 5.3^a^	47.2 ± 3.2^a^	61.4 ± 2.1^a^	57.1 ± 4.1	56.3 ± 1.4^a^	ns	ns	ns
(-) epicatechin^1^	39.1 ± 2.4^ab^	38.9 ± 1.0^abc^	31.8 ± 1.5^de^	26.6 ± 0.4^ef^	40.7 ± 2.7^a^	34.5 ± 2.6^bcd^	24.3 ± 0.6^f^	23.9 ± 1.1^f^	32.6 ± 2.2^d^	32.7 ± 0.9^d^	33.8 ± 1.2 ^cd^	25.2 ± 2.5^f^	***	***	***
***Total***	**299.1 ± 16.3^abc^**	**259.7 ± 13.5^de^**	**268.3 ± 27.1^cde^**	**264.9 ± 11.0^cde^**	**322.9 ± 1.1^a^**	**309.3 ± 9.7^abc^**	**231.1 ± 13.6^e^**	**272.3 ± 18.8^bcde^**	**317.5 ± 28.3^ab^**	**298.0 ± 10.3^abcd^**	**236.4 ± 4.5^e^**	**269.9 ± 1.3^cde^**	***	ns	**
**Stilbenes (mg/L)**															
*trans*-piceid^6^	nd	nd	nd	nd	2.1 ± 0.01^a^	2.0 ± 0.1^ab^	1.7 ± 0.1^ab^	1.1 ± 0.4^c^	1.8 ± 0.2^ab^	2.0 ± 0.1^ab^	1.7 ± 0.1^b^	1.9 ± 0.01^ab^	***	***	***
*trans*-Resveratrol^1^	0.9 ± 0.05^ef^	1.0 ± 0.1^ef^	0.7 ± 0.04^f^	0.6 ± 0.03^f^	2.2 ± 0.1^ab^	2.4 ± 0.1^a^	1.3 ± 0.03^de^	1.7 ± 0.4^cd^	1.8 ± 0.4^bc^	2.2 ± 0.02^abc^	1.3 ± 0.02^e^	1.2 ± 0.1^e^	***	***	**
***Total***	**0.9 ± 0.05^d^**	**1.0 ± 0.08^d^**	**0.7 ± 0.04^d^**	**0.6 ± 0.03^d^**	**4.3 ± 0.1^ab^**	**4.4 ± 0.09^a^**	**3.0 ± 0.1^c^**	**2.8 ± 0.7^c^**	**3.6 ± 0.6^bc^**	**4.1 ± 0.1^ab^**	**2.9 ± 0.09^c^**	**3.1 ± 0.1^c^**	***	***	**
***TOTAL***	**1386.2 ± 81.3^abc^**	**1504.0 ± 38.5^a^**	**1365.7 ± 100.3^abc^**	**1300.3 ± 44.6 ^cd^**	**1466.1 ± 13.5^ab^**	**1363.5 ± 12.3^abc^**	**1139.2 ± 17.4^e^**	**1056.0 ± 66.4^e^**	**1396.2 ± 38.9^abc^**	**1339.7 ± 19.6^bc^**	**1105.3 ± 10.5^e^**	**1160.4 ± 13.2^de^**	***	***	***

^†^In row, different letters indicate statistically significant differences at *p* < 0.05 according to two-way ANOVA with interaction followed by Tukey’s test. Significance: ns, *, **, and ***, not significant or significant at *p* < 0.05, *p* < 0.01, or *p* < 0.001, respectively. Average value ± standard deviation (n = 3). *Abbreviations*: C, control wine; I, wine with infusion of vine-shoot chips; U + I, wine with ultrasound and infusion of vine-shoot chips; nd, not detected; De-3-glc, Delphinidin 3-glucoside; Pet-3-glc, Petunidin 3-glucoside; Peo-3-glc, Peonidin 3-glucoside; Mav-3-glc, Malvidin 3-glucoside; VitA, Vitisin A; VitB, Vitisin B; Mv-3-O-glc-(epi)cat, Malvidin-3-O-glucoside-(epi)cathechin; Pet-3-(6′'-act)-glc, Petunidin-3-(6′'-acetyl)-glucoside; Peo-3-(6′'-act)-glc, Peonidin-3-(6′'-acetyl)-glucoside; Mv-3-(6′'-act)-glc, Malvidin 3-(6′'-acetyl)-glucoside; Pet-3-(6′'-t-cm)-glc, Petunidin 3-(6′'-t-coumaroyl)-glucoside; Mv-3-(6′'-c-cm)-glc, Malvidin 3-(6′'-c-coumaroyl)-glucoside; Peo-3-(6′'-t-cm)-glc, Peonidin 3-(6′'-t-coumaroyl)-glucoside; Mv-3-(6′'-t-cm)-glc, Malvidin 3-(6′'-t-coumaroyl)-glucoside; Q3OG, Quercetin 3-O-glucuronide.

1 Quantified using corresponding standards; ^2^expressed as malvidin 3-glucoside equivalents; ^3^expressed as caftaric acid; ^4^expressed as quercetin; ^5^expressed as (+) catechin; ^6^expressed as *trans*-Resveratrol.

The most abundant anthocyanin was, as expected, malvidin 3-glucoside. The anthocyanins total content was dependent on the time and the treatment applied (*p* < 0.001). In fact, all wines had similar concentrations after 7 days, but the concentration was higher in the control wine after 14 days. After 28 days, the concentration decreased in wines I and U + I by 40 % and 33 %, respectively, showing a similar trend than anthocyanins quantified by spectrophotometry. Although with different reduction percentages, these results are in agreement with previous studies in which the effect of wood chips on phenolic wine composition was evaluated [30], [32], [57], [61]. This decrease could be explained by the adsorption of anthocyanins on vine-shoot chips [62]. No substantial effects of the ultrasound treatment were observed in the anthocyanin profile compared to chips infusion.

The total phenolic acids concentration was dependent on the sampling time (*p* < 0.001) and the treatment applied (*p* < 0.05). After 7 and 14 days, all wines had a similar concentration, but after 28 days the concentration decreased in wines C and I and increased in wine U + I (+10 %, compared to day 7). The increase was correlated to the higher concentration in gallic acid. Extraction of gallic acid from chips was therefore enhanced by ultrasound treatment [63].

Flavonols, a group of compounds in the flavonoid family, included quercetin 3-O-glucuronide, myricetin and quercetin. Their total concentration was affected by treatment (*p* < 0.001) and sampling time (*p* < 0.001): they decreased in the experimental wine after the addition of chips, decreased in all wine during storage and eventually disappeared from the aged wines as in the case of myricetin and quercetin for I and U + I wine at 21 and 28 days of sampling. Literature reports increases of flavonols after contact with oak chips. These contrasting results could be related to the different botanical origin of the chips used in this research [64].

All wines had a similar concentration of total flavanols, but after 14 days the concentration decreased. In terms of single compounds, procyanidin B1 was the most abundant compound and remained constant after each treatment, though decreasing during contact time.

The total stilbenes concentration and the stilbenic composition were dependent on the treatment applied and the treatment time (*p* < 0.001): I and U + I wines had higher total stilbene concentration than C wines, but the concentration decreased during the contact with chips (after 14 days). Specifically, the only stilbenic compounds quantified in wines were *trans*-piceid and *trans*-resveratrol. The first one was present only in I and U + I samples in similar concentrations, the second one increased with the addition of toasted vine-shoots chips (with and without ultrasound treatment) if compared with control wines. Similar concentrations of *trans*-resveratrol were found in a previous study [31] which observed that the addition of vine-shoots made a significant increase of this compound to wines, independent of the time of addition during the winemaking process. In fact, several authors have demonstrated that vine-shoots are a source of stilbenes (especially *trans*-resveratrol) [65], [66]. Moreover, their beneficial effects on human health related to wine consumption were widely discussed [67], [68].

### 3.4 Volatile compounds

A total of 49 compounds were identified in the whole set of samples and divided into nine chemical groups: alcohols (12), aldehydes (7), acetate esters (7), ethyl esters (11), other esters (2), ketones (2), terpenes (2), noroisoprenoids (1), carboxylic acids (5) (Table S1). Among them thirteen compounds exhibited OAVs greater than one (Table S2).

**Fig. 1:**
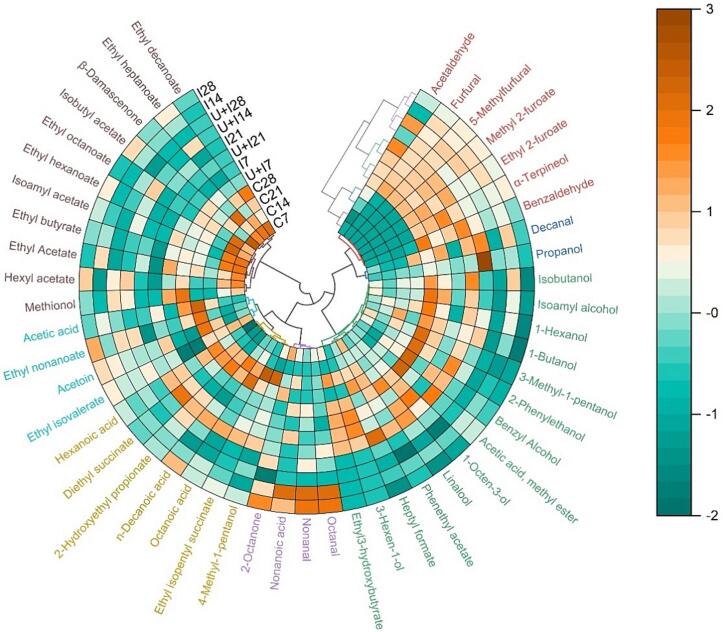
Polar heatmap with a circular dendrogram deriving from a hierarchical cluster analysis of the volatile profiles of the wine samples. Abbreviations: C, control wine; I, wine with infusion of vine-shoot chips; U + I, wine with ultrasound and infusion of vine-shoot chips. The sampling time (days) is indicated by the figures after abbraviations (7, 14, 21, 28).

Fig. 1 reports a polar heatmap with a circular dendrogram obtained from a hierarchical cluster analysis. Hierarchical clustering highlights groups with similar behaviour among both wines and volatile compounds [69]. Clustering showed that the wines treated with toasted vine-shoot chips and ultrasound had volatile profiles different from control wine. In fact, four clusters were obtained for wine samples (marked with red, blue, green, and violet colour). The volatile compounds were instead clustered in seven groups (marked with red, blue, green, violet, yellow, turquoise, brown colour looking at clockwise the circular heatmap). The first cluster (analysing clockwise the circular heatmap) included four aldehydes, two esters and one terpene. This cluster characterized the wines obtained by only chips infusion and ultrasonic treatment and chips infusion. Particular attention was given to the class of aldehydes. Probably, these compounds were transferred into wines because of contact with toasted vine-shoot chips. In particular, furfural and 5-methylfurfural are two furan compounds generated during the toasting process of the chips, which determined in wine toasted almond and caramel aromas [70]. The first originates from pentose compounds of hemicelluloses, the second one derives from hexoses present in cellulose [71]. Furfural was detected in control wine at very low concentration, while it was higher in the treated wines although decreased during time (Table S1). In fact, once furfural has been extracted from toasted vine-shoots chips, can be involved in different reaction which occur in wine over time [72], [73], [74]. Both furfural and 5-methylfurfural have been found in wines treated with chips of different botanical origin [75], [76]. The volatile compounds belonging to this first cluster included benzaldehyde, which is responsible of bitter almond-like notes [77], methyl-2-furoate (fruity notes) [78], ethyl-2-furoate (balsamic notes) [79] and α-terpineol (sweet, floral notes) [80]. These compounds, despite having an OAV < 1, could contribute to the wine aroma through an additive effect with other similar compounds [81]. The second and third cluster contained 10 alcohols, 1 aldehyde, 4 esters and 1 terpene. Most of alcohols were contained at higher concentration in wine samples treated with chips (I wines after 21 days and U + I wines after 7 days of contact with chips). Alcohols are wine volatile fermentative compounds which could positively influence the wine aromatic profile, increasing the fruity and flowery notes [82]. Among them, 2-phenylethanol and 1-octen-3-ol had an odour OAV > 1. Moreover, other compounds of these cluster had an OAV > 1: linalool which characterized the wines I and U + I after 7 and 21 days of treatment; decanal which was particularly present in control wines after 28 days of contact, in I wines up to 21 days of treatment and U + I up to 14. The fourth cluster contained 4 volatile compounds that characterized wines treated with toasted vine-shoot chips after 14 and 28 days of contact. Among them, the compounds with an OAV > 1 have been related to perceptions of green (octanal) or green and citrus (nonanal) [83], [84]. Finally, the last three clusters particularly characterized the wines control and contained most of esters (ethyl and acetate esters). Esters are related to fruity and floral notes in young wines. Their fermentative origin is linked to amino acid metabolism in the case of acetates, and fatty acid metabolism for ethyl esters [85], [86], [87]. The total concentration of ethyl esters and acetate esters is affected by both treatments and time (Table S1). The control wine had the highest total concentration of these compounds, on the other hand, treated wines showed a decreasing trend during time. This reduction might have been caused by their hydrolysis [88]. Moreover, the wood chips added could absorb esters, resulting in a decrease in the content of these compounds, as reported in previous studies [88], [89], [90]. In these clusters, isoamyl acetate, ethyl butyrate, ethyl isovalerate, ethyl hexanoate, ethyl heptanoate, ethyl octanoate, all linked to fruity notes had an OAV > 1. The other compounds found in these clusters have been related to perception such as fruit and sweet (β-damascenone, OAV > 1) and fruity, cheese, fatty, and green notes (acetic acid, hexanoic acid, octanoic acid, decanoic acid) [91], [92], [93].

### 3.5 Sensory analysis

**Fig. 2:**
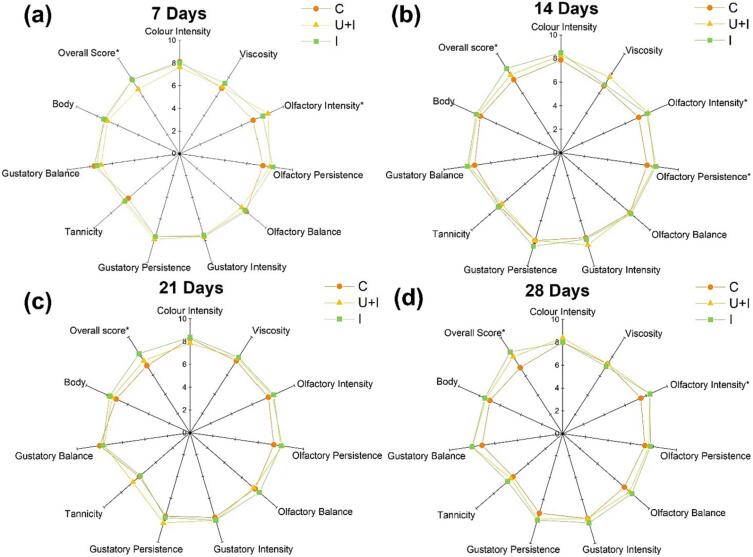
Incidence of treatment and sampling time on sensory profile of control wine (C), wine treated with ultrasound and infusion of toasted vine-shoot chips (U + I), wine with infusion of toasted vine-shoot chips (I) after 7 (a), 14 (b), 21 (c) and 28 (d) sampling days.*Sensory parameters where there is significantly difference among the wines (Tukey test, *p* < 0.05). Abbreviations: C, control wine; I, wine with infusion of vine-shoot chips; U + I, wine with ultrasound and infusion of vine-shoot chips.

The analysis of variance did not show significant differences among the tested ageing modes in any time, except for olfactory intensity and persistence and overall score (Fig. 2). After 7 days of treatment (Fig. 2a), wine U + I had higher olfactory intensity than wines I and C. These latter had obtained a higher score than U + I for the overall score. After 14 days (Fig. 2b), wine C exhibited less olfactory intensity and persistence than the other wines. Finally, after 28 days of treatment, wines I and U + I had higher olfactory intensity than C. Statistically significant differences among the wines were obtained for the overall score: after 14, 21 and 28 days of treatment, wine I received the highest score, followed by U + I and C (Fig. 2b, 2c, 2d).

**Fig. 3:**
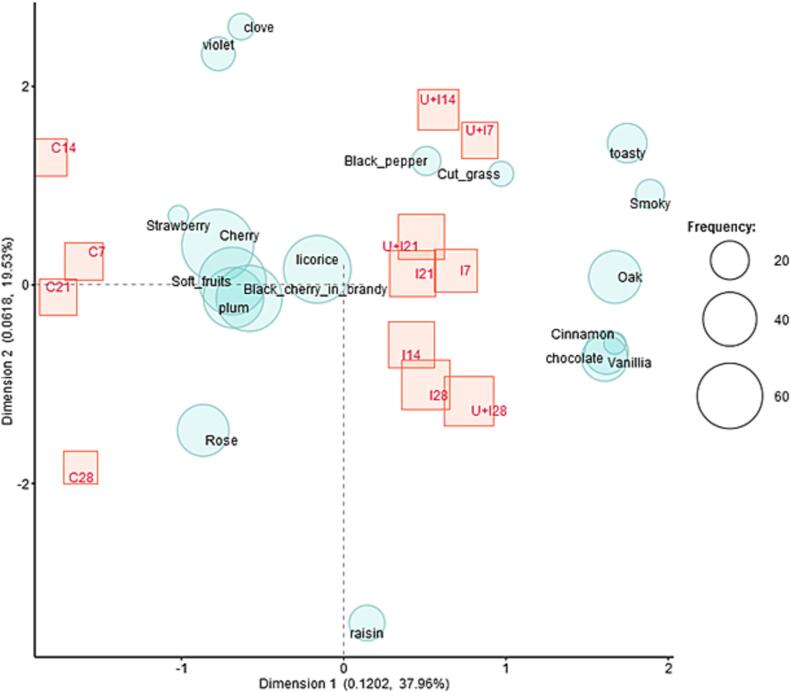
Correspondence analysis of aroma descriptors of control wine (C), wine with infusion of toasted vine-shoot chips (I), wine with ultrasound and infusion of toasted vine-shoot chips (U + I) after 7, 14, 21 and 28 sampling days.

Wine olfactory profile was also valuated by check-all-that-apply (CATA) approach. The assessment of the selected aroma descriptors was performed via smell and represented by correspondence analysis, displayed in Fig. 3. Soft fruits, cherry and liquorice notes were highly appreciated in all wines at any of the moments. Up to 28 days of treatment, fragrance notes of chocolate, toasted, oak, smoky and vanilla were perceived only in wines treated with toasted vine-shoots chips, with and without ultrasounds. The perception of these notes related to wood could possibly have been due to the toasting vine-shoots chips added, since these aromas come from compounds generated during the wood toasting (such as furanic compounds) [36], [94] and they are typical of wines aged in barrels or with wood chips [28], [74], [76]. The effect of ultrasound on sensory properties could be observed for shorter contact times (7–14 days), conferring green, black pepper, smoky notes to wines.

**Fig. 4:**
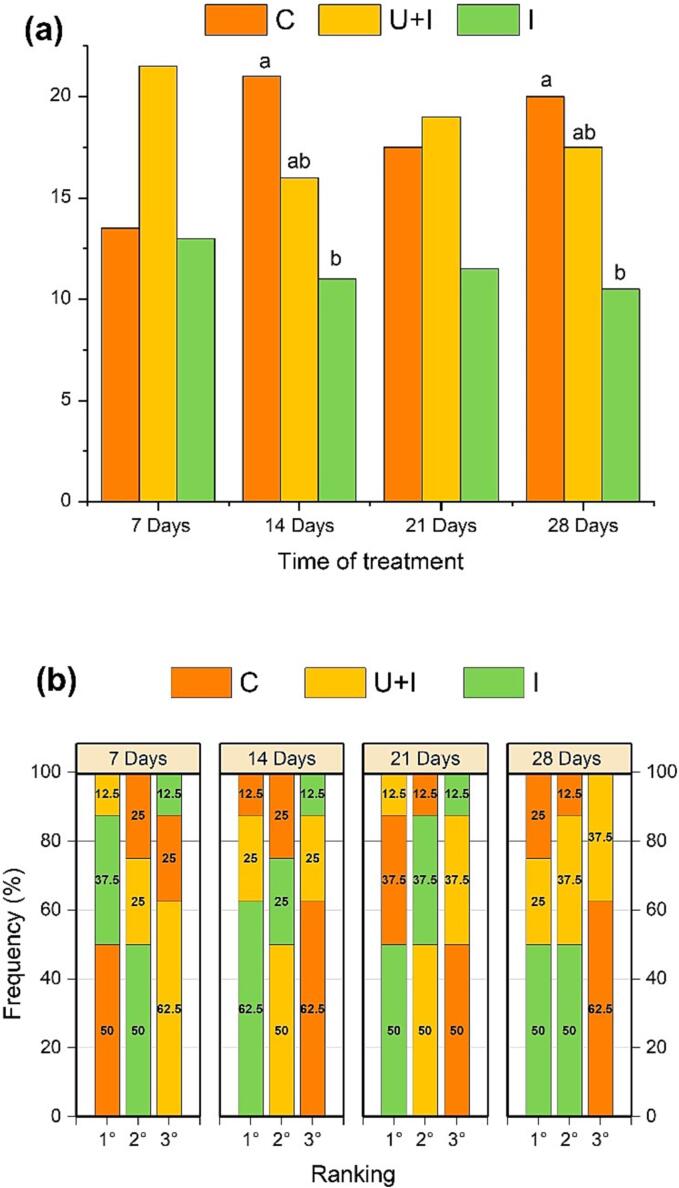
Global scores (sum of rankings) (a) and percentage of tasters who have rated in descending rank order of preference (from 1° to 3°) (b) each wine samples, after each time of treatment. Abbreviations: C, control wine; I, wine with infusion of vine-shoot chips; U + I, wine with ultrasound and infusion of vine-shoot chips.

The global scores of tasters’ preferences for each wine are shown in Fig. 4a. According to the process used, lower ranking values correspond to higher judges’ preferences. Significant differences were found among the wine samples only after 14 and 28 days of treatment, using Friedman’s test. These results can be explained by the fact that “preference” is a subjective concept: certain characteristics (such as wood notes) were considered as positive by some judges but as negative by others. Therefore, Fig. 4b shows in detail the percentage of tasters who had chosen each wine in first, second or third place by preference. After 7 days, wine C was chosen in first place (i.e., the most preferred wine) by 50 % of tasters and in third place (i.e., the least preferred wine) by 25 % of tasters, whereas wine U + I was placed as third by 62.5 % of tasters. Differently, after 14 days, 62.5 % of tasters preferred wine I (first place), followed by U + I and C. Even after 21 days, wine I was chosen in first place by 50 % of tasters, in second by 37.5 % of tasters and in third only by 12.5 %. After 28 days, 50 % of the tasters continued to prefer wine I (first and second place, 50 %), placing C wine in the last place (62.5 %).

## 4 Conclusions

Traditional ageing of wines is a complex process in which several chemical reactions take place and, frequently, the long-time of storage is incompatible with market requirements and represents a high cost for wineries. The implementation of new techniques able to accelerate the process while ensuring the wine quality is highly demanded. Ultrasound technology combined with the use of wood chips could be a promising technology to reduce ageing times. The main results of this work applied to Primitivo wine demonstrated that the combination of ultrasound with addition of vine-shoot chips can have a significant influence on the phenolic composition, favouring tannin evolution that could accelerate the ageing process. Also, olfactory properties can be tuned by ultrasound treatment, when combined with shorter contact with chips. On the other hand, longer contact times with toasted vine-shoot chips could determine an enrichment of compounds associated with new wood-related aromas (i.e., furfural, 5-methylfurfural, benzaldehyde, methyl-2-furoate, ethyl-2-furoate) and notes of chocolate, toasted, oak or vanilla which were positively considered by the tasters involved in the study.

According to the results obtained, vine-shoots could be considered a viticultural waste that may be turned into an oenological coadjuvant if appropriately treated with ultrasound and used during wine ageing. In this case, they would acquire a new life and provide enormous management and economic advantages for wineries.

## Ethical statement

Participants gave informed consent via the statement “I am aware that my responses are confidential, and I agree to participate in this survey” where an affirmative reply was required to enter the survey. They were able to withdraw from the survey at any time without giving a reason. The products tested were safe for consumption.

## CRediT authorship contribution statement

**Mirella Noviello:** Writing – original draft, Visualization, Methodology, Investigation. **Vito Michele Paradiso:** Writing – review & editing, Visualization, Supervision, Methodology, Conceptualization. **Giuseppe Natrella:** Writing – review & editing, Formal analysis, Data curation. **Giuseppe Gambacorta:** Writing – review & editing, Formal analysis, Data curation. **Michele Faccia:** Writing – review & editing, Validation, Resources. **Francesco Caponio:** Writing – review & editing, Supervision.

## Declaration of competing interest

The authors declare that they have no known competing financial interests or personal relationships that could have appeared to influence the work reported in this paper.

## Data Availability

Data will be made available on request.

## References

[1] Ma T., Wang J., Wang H., Zhao Q., Zhang F., Ge Q., Li C., Gutiérrez-Gamboa G., Fang Y., Sun X. (2022). Wine aging and artificial simulated wine aging: technologies, applications, challenges, and perspectives. Food Res. Int..

[2] Bhargava N., Mor R.S., Kumar K., Sharanagat V.S. (2021). Advances in application of ultrasound in food processing: a review. Ultrason. Sonochem..

[3] Fu X., Belwal T., Cravotto G., Luo Z. (2020). Sono-physical and sono-chemical effects of ultrasound: primary applications in extraction and freezing operations and influence on food components. Ultrason. Sonochem..

[4] Gambacorta G., Trani A., Punzi R., Fasciano C., Leo R., Fracchiolla G., Faccia M. (2017). Impact of ultrasounds on the extraction of polyphenols during winemaking of red grapes cultivars from southern Italy. IFSET.

[5] Maza M., Álvarez I., Raso J. (2019). Thermal and non-thermal physical methods for improving polyphenol extraction in red winemaking. Beverages.

[6] Pérez-Porras P., Bautista-Ortín A.B., Jurado R., Gómez-Plaza E. (2021). Using high-power ultrasounds in red winemaking: effect of operating conditions on wine physico-chemical and chromatic characteristics. LWT.

[7] Pérez-Porras P., Bautista-Ortín A.B., Jurado R., Gómez-Plaza E. (2022). Combining high-power ultrasound and enological enzymes during winemaking to improve the chromatic characteristics of red wine. LWT.

[8] Organisation International de la Vigne et du Vin (OIV), Resolution OIV-OENO 616-2019, Tretament of Crushed Grapes with Ultrasound to Promote the Extraction of Their Compounds Resolution. Retrieved March 17, 2023, from https://www.oiv.int/public/medias/6826/oiv-oeno-616-2019-en.pdf.

[9] Silva F.V.M., van Wyk S. (2021). Emerging non-thermal technologies as alternative to SO_2_ for the production of wine. Foods.

[10] Cacciola V., Batllò I.F., Ferraretto P., Vincenzi S., Celotti E. (2013). Study of the ultrasound effects on yeast lees lysis in winemaking. Eur. Food Res. Technol..

[11] del Fresno J.M., Loira I., Morata A., González C., Suárez-Lepe J.A., Cuerda R. (2018). Application of ultrasound to improve lees ageing processes in red wines. Food Chem..

[12] Kulkarni P., Loira I., Morata A., Tesfaye W., González M.C., Suárez-Lepe J.A. (2015). Use of non-saccharomyces yeast strains coupled with ultrasound treatment as a novel technique to accelerate ageing on lees of red wines and its repercussion in sensorial parameters. LWT.

[13] Osete-Alcaraz A., Bautista-Ortín A.B., Pérez-Porras P., Gómez-Plaza E. (2021). The application of ultrasound and enzymes could be promising tools for recovering polyphenols during the aging on lees process in red winemaking. Foods.

[14] Aliaño-González M.J., Richard T., Cantos-Villar E. (2020). Grapevine cane extracts: raw plant material, extraction methods, quantification, and applications. Biomolecules.

[15] F.J. Barba, Z. Zhu, M. Koubaa, A.S. Sant’Ana, V. Orlien, Green alternative methods for the extraction of antioxidant bioactive compounds from winery wastes and by-products: A review, Trends Food Sci. Technol. 49 (2016), 96–109. https://doi.org/10.1016/j.tifs.2016.01.006.

[16] Dorosh O., Moreira M.M., Rodrigues F., Peixoto A.F., Freire C., Morais S., Delerue-Matos C. (2020). Vine-canes valorisation: ultrasound-assisted extraction from lab to pilot scale. Molecules.

[17] González M., Barrios S., Budelli E., Pérez N., Lema P., Heinzen H. (2020). Ultrasound assisted extraction of bioactive compounds in fresh and freeze-dried Vitis vinifera cv tannat grape pomace. Food Bioprod. Process..

[18] Piñeiro Z., Marrufo-Curtido A., Serrano M., Palma M. (2016). Ultrasound-assisted extraction of stilbenes from grape canes. Molecules.

[19] Celotti E., Stante S., Ferraretto P., Román T., Nicolini G., Natolino A. (2020). High power ultrasound treatments of red young wines: effect on anthocyanins and phenolic stability indices. Foods.

[20] García Martín J.F., Sun D.W. (2013). Ultrasound and electric fields as novel techniques for assisting the wine ageing process: the state-of-the-art research. Trends Food Sci. Technol..

[21] Solar S., Castro R., Guerrero E.D. (2021). New accelerating techniques applied to the ageing of oenological products. Food Rev. Int..

[22] Cárcel J.A., García-Pérez J.V., Benedito J., Mulet A. (2012). Food process innovation through new technologies: use of ultrasound. J. Food Eng..

[23] Organisation International de la Vigne et du Vin (OIV), Resolution OENO 9/2001, Usage of pieces of oak wood in winemaking, in International Codex of Oenological Practices. Office International de la Vigne et du Vin: Paris, France, 2001.

[24] European Union. Commission, Regulation (EC) No 1507/2006, in Official Journal of the European Union 2006, 280, 1-9.

[25] Costa M., Fontes L., Correia A.C., Miljić U., Jordão A.M. (2020). Impact of oak (Q. pyrenaica and Q. pubescens) and cherry (P. avium) wood chip contact on phenolic composition and sensory profile evolution of red wines during bottle storage. OENO One.

[26] Del Galdo V., Correia A.C., Jordão A.M., da Silva J.M.R. (2019). Blends of wood chips from oak and cherry: impact on the general phenolic parameters and sensory profile of a white wine during the aging process. Vitis.

[27] Jordão A.M., Cosme F. (2022). The application of wood species in enology: chemical wood composition and effect on wine quality. Appl. Sci..

[28] Lisanti M.T., Capuano R., Moio L., Gambuti A. (2021). Wood powders of different botanical origin as an alternative to barrel aging for red wine. Eur. Food Res. Technol..

[29] Cebrián-Tarancón C., Sánchez-Gómez R., Carot J.M., Zalacain A., Gonzalo L.A., Salinas M.R. (2019). Assessment of vine-shoots in a model wines as enological additives. Food Chem..

[30] Cebrián-Tarancón C., Sánchez-Gómez R., Cabrita M.J., García R., Zalacain A., Alonso G.L., Salinas M.R. (2019). Winemaking with vine-shoots. modulating the composition of wines by using their own resources. Food Res. Int..

[31] Cebrián-Tarancón C., Fernández-Roldán F., Sánchez-Gómez R., Alonso G.L., Salinas M.R. (2022). Pruned vine-shoots as a new enological additive to differentiate the chemical profile of wines. Food Res. Int..

[32] Fanzone M., Catania A., Assof M., Jofré V., Prieto J., Gil Quiroga D., Lacognata Sottano J., Sari S. (2021). Application of vine-shoot chips during winemaking and aging of malbec and bonarda wines. Beverages.

[33] Sánchez-Córdoba C., Durán-Guerrero E., Castro R. (2021). Olfactometric and sensory evaluation of red wines subjected to ultrasound or microwaves during their maceration or ageing stages. LWT.

[34] Tao Y., Zhang Z., Sun D.W. (2014). Experimental and modeling studies of ultrasound-assisted release of phenolics from oak chips into model wine. Ultrason. Sonochem..

[35] Cebrián C., Sánchez-Gómez R., Salinas M.R., Alonso G.L., Zalacain A. (2017). Effect of post-pruning vine-shoots storage on the evolution of high-value compounds. Ind. Crops Prod..

[36] Cebrián-Tarancón C., Sánchez-Gómez R., Salinas M.R., Alonso G.L., Oliva J., Zalacain A. (2018). Toasted vine-shoot chips as enological additive. Food Chem..

[37] Gambacorta G., Faccia M., Trani A., Lamacchia C., Gomes T. (2012). Phenolic composition and antioxidant activity of southern italian monovarietal virgin olive oils. Eur. J. Lipid Sci. Technol..

[38] Gambacorta G., Antonacci D., La Gatta M., Faccia M., La Gatta B., Pati S., Coletta A., La Notte E. (2011). Phenolic composition of aglianico and nero di troia grapes and wines as affected by cover cropping and irrigation. Ital. J. Food Sci..

[39] Glories Y. (1984). La couleur des vins rouges. Conn. Vigne Vin.

[40] Tarantino A., Difonzo G., Lopriore G., Disciglio G., Paradiso V.M., Gambacorta G., Caponio F. (2020). Bioactive compounds and quality evaluation of ‘wonderful’ pomegranate fruit and juice as affected by deficit irrigation. J. Sci. Food Agric..

[41] Torreggiani A., Demarinis C., Pinto D., Papale A., Difonzo G., Caponio F., Pontonio E., Verni M., Rizzello C.G. (2023). Up-cycling grape pomace through sourdough fermentation: characterization of phenolic compounds, antioxidant activity, and anti-inflammatory potential. Antioxidants.

[42] Ginjom I., D’Arcy B., Caffin N., Gidley M. (2011). Phenolic compound profiles in selected Queensland red wines at all stages of the wine-making process. Food Chem..

[43] Marquez A., Dueñas M., Serratosa M.P., Merida J. (2013). Identification by HPLC-MS of anthocyanin derivatives in raisins. J. Chem*.*.

[44] Pérez-Navarro J., Izquierdo-Cañas P.M., Mena-Morales A., Martínez-Gascueña J., Chacón-Vozmediano J.L., García-Romero E., Hermosín-Gutiérrez I., Gómez-Alonso S. (2019). Phenolic compounds profile of different berry parts from novel *Vitis vinifera* L. red grape genotypes and tempranillo using HPLC-DAD-ESI-MS/MS: a varietal differentiation tool. Food Chem..

[45] Topi D., Kelebek H., Guclu G., Selli S. (2022). LC-DAD-ESI-MS/MS characterization of phenolic compounds in wines from Vitis vinifera ‘shesh i bardhë’ and ‘vlosh’ cultivars. J. Food Process. Preserv..

[46] Zhang X.K., Li S.Y., Zhao X., Pan Q.H., Shi Y., Duan C.Q. (2020). HPLC-MS/MS-based targeted metabolomic method for profiling of malvidin derivatives in dry red wines. Food Res. Int..

[47] I. Prezioso, G. Fioschi, L. Rustioni, M. Mascellani, G. Natrella, P. Venerito, G. Gambacorta, V.M., V.M. Paradiso, Influence of prolonged maceration on phenolic compounds, volatile profile and sensory properties of wines from Minutolo and Verdeca, two Apulian white grape varieties, LWT 192 (2024), 115698. https://doi.org/10.1016/j.lwt.2023.115698.

[48] Iland P., Bruer D., Bruer N., Edwards G., Ewart A., Ford C., Markides A., Sitters J., Wilkes E., Caloghiris S. (2021).

[49] Meyners M., Castura J.C., Carr B.T. (2013). Existing and new approaches for the analysis of CATA data. Food Qual. Prefer..

[50] Zhang Q.A., Shen Y., Fan X., Martín J.F.G., Wang X., Song Y. (2015). Free radical generation induced by ultrasound in red wine and model wine: an EPR spin-trapping study. Ultrason. Sonochem..

[51] Q.A. Zhang, B.W. Xu, B.Y Chen., W.Q. Zhao, C.H. Xue. Ultrasound as an effective technique to reduce higher alcohols of wines and its influencing mechanism investigation by employing a model wine, Ultrason. Sonochem. 61 (2020), 104813. . 10.1016/j.ultsonch.2019.104813 31670251

[52] Zhang Q.A., Wang T.T. (2017). Effect of ultrasound irradiation on the evolution of color properties and major phenolic compounds in wine during storage. Food Chem..

[53] Rossetti F., Jouin A., Jourdes M., Teissedre P.L., Foligni R., Longo E., Boselli E. (2020). Impact of different stoppers on the composition of red and rosé lagrein, schiava (vernatsch) and merlot wines stored in bottle. Molecules.

[54] P. Ribéreau-Gayon, Y. Glories, A. Maujean, D. Dubourdieu, I composti fenolici, in: Trattato di enologia 2, Chimica del vino, stabilizzazione e trattamenti, fourth ed., Edagricole, Milano, 2019, pp 194-196.

[55] M.T. Escribano-Bailón, J.C. Rivas-Gonzalo, I. García-Estévez, Wine color evolution and stability, in: red wine technology, Academic Press, 2019, pp. 195–205. https://doi.org/10.1016/B978-0-12-814399-5.00013-X.

[56] Jordão A.M., Lozano V., González-SanJosé M.L. (2019). Influence of different wood chip extracts species on color changes and anthocyanin content in synthetic wine solutions. Foods.

[57] Costa M., Miglior N., Correia A.C., Ricardo-da-Silva J.M., Jordão A.M. (2021). Storage of a touriga nacional red wine in contact with *Juglans regia* L. and *Quercus petraea* L. wood chip species: comparative influence on phenolic and sensory characteristics. Eur. Food Res. Technol..

[58] Laqui-Estaña J., López-Solís R., Peña-Neira Á., Medel-Marabolí M., Obreque-Slier E. (2019). Wines in contact with oak wood: the impact of the variety (carménère and cabernet sauvignon), format (barrels, chips and staves), and aging time on the phenolic composition. J. Sci.food Agric..

[59] Suriano S., Alba V., Tarricone L., Di Gennaro D. (2015). Maceration with stems contact fermentation: effect on proanthocyanidins compounds and color in primitivo red wines. Food Chem..

[60] Platzer M., Kiese S., Herfellner T., Schweiggert-Weisz U., Miesbauer O., Eisner P. (2021). Common trends and differences in antioxidant activity analysis of phenolic substances using single electron transfer based assays. Molecules.

[61] del Álamo Sanza M., Nevares Domínguez I., García Merino S. (2004). Influence of different aging systems and oak woods on aged wine color and anthocyanin composition. Eur. Food Res. Technol..

[62] Barrera-García V.D., Gougeon R.D., Di Majo D., De Aguirre C., Voilley A., Chassagne D. (2007). Different sorption behaviors for wine polyphenols in contact with oak wood. J. Agric. Food Chem..

[63] M. del Álamo Sanza, I. Nevares Domínguez, L.M. Cárcel Cárcel, L. Navas Gracia, Analysis for low molecular weight phenolic compounds in a red wine aged in oak chips, Anal. Chim. Acta. 513 (2004), 229–237. https://doi.org/10.1016/j.aca.2003.11.041.

[64] Baiano A., De Gianni A., Mentana A., Quinto M., Centonze D., Del Nobile M.A. (2016). Effects of the treatment with oak chips on color-related phenolics, volatile composition, and sensory profile of red wines: the case of aglianico and montepulciano. Eur. Food Res. Technol*.*.

[65] Noviello M., Caputi A.F., Squeo G., Paradiso V.M., Gambacorta G., Caponio F. (2022). Vine shoots as a source of *trans*-resveratrol and *ε*-viniferin: a study of 23 italian varieties. Foods.

[66] Sánchez-Gómez R., Zalacain A., Alonso G.L., Salinas M.R. (2016). Effect of toasting on non-volatile and volatile vine-shoots low molecular weight phenolic compounds. Food Chem..

[67] Benbouguerra N., Hornedo-Ortega R., Garcia F., El Khawand T., Saucier C., Richard T. (2021). Stilbenes in grape berries and wine and their potential role as anti-obesity agents: a review. Trends Food Sci. Technol..

[68] Nemzer B., Kalita D., Yashin A.Y., Yashin Y.I. (2021). Chemical composition and polyphenolic compounds of red wines: their antioxidant activities and effects on human health—A review. Beverages.

[69] Paradiso V.M., Sanarica L., Zara I., Pisarra C., Gambacorta G., Natrella G., Cardinale M. (2022). Cultivar-dependent effects of non-saccharomyces yeast starter on the oenological properties of wines produced from two autochthonous grape cultivars in southern Italy. Foods.

[70] Flamini R., Panighel A., De Marchi F. (2021). Mass spectrometry in the study of wood compounds released in the barrel-aged wine and spirits. Mass Spectrom. Rev..

[71] Dumitriu G.D., Teodosiu C., Gabur I., Cotea V.V., Peinado R.A., López de Lerma N. (2019). Evaluation of aroma compounds in the process of wine ageing with oak chips. Foods.

[72] Nonier Bourden M.N., Vivas N., Absalon C., Vitry C., Fouquet E., De Gaulejac N.V. (2008). Structural diversity of nucleophilic adducts from flavanols and oak wood aldehydes. Food Chem..

[73] Fernández De Simón B., Martínez J., Sanz M., Cadahía E., Esteruelas E., Muñoz A.M. (2014). Volatile compounds and sensorial characterisation of red wine aged in cherry, chestnut, false acacia, ash and oak wood barrels. Food Chem..

[74] Rubio-Bretón P., Garde-Cerdán T., Martínez J. (2018). Use of oak fragments during the aging of red wines. effect on the phenolic, aromatic, and sensory composition of wines as a function of the contact time with the wood. Beverages.

[75] Dumitriu G.D., Peinado R.A., Cotea V.V., López de Lerma N. (2020). Volatilome fingerprint of red wines aged with chips or staves: influence of the aging time and toasting degree. Food Chem..

[76] Santos F., Correia A.C., Ortega-Heras M., García-Lomillo J., González-SanJosé M.L., Jordão A.M., Ricardo-da-Silva J.M. (2019). Acacia, cherry and oak wood chips used for a short aging period of rosé wines: effects on general phenolic parameters, volatile composition and sensory profile. J. Sci. Food Agric..

[77] Mayr C.M., Capone D.L., Pardon K.H., Black C.A., Pomeroy D., Francis I.L. (2015). Quantitative analysis by GC-MS/MS of 18 aroma compounds related to oxidative off-flavor in wines. J. Agric. Food Chem..

[78] Piombino P., Moio L., Genovese A. (2019). Orthonasal vs. retronasal: studying how volatiles’ hydrophobicity and matrix composition modulate the release of wine odorants in simulated conditions. Food Res. Int..

[79] Wang N., Chen S., Zhou Z. (2019). Characterization of volatile organic compounds as potential aging markers in chinese rice wine using multivariable statistics. J. Sci. Food Agric..

[80] Genovese A., Basile B., Lamorte S.A., Lisanti M.T., Corrado G., Lecce L., Strollo D., Moio L., Gambuti A. (2021). Influence of berry ripening stages over phenolics and volatile compounds in aged aglianico wine. Horticulturae.

[81] Francis I.L., Newton J.L. (2005). Determining wine aroma from compositional data. Aust. J. Grape Wine Res..

[82] Liu J., Zhao W., Li S., Zhang A., Zhang Y., Liu S. (2018). Characterization of the key aroma compounds in proso millet wine using headspace solid-phase microextraction and gas chromatography-mass spectrometry. Molecules.

[83] Guo X., Ho C.T., Wan X., Zhu H., Liu Q., Wen Z. (2021). Changes of volatile compounds and odor profiles in Wuyi rock tea during processing. Food Chem..

[84] J., Song, Y. Shao, Y. Yan, X. Li, , J. Peng & L. Guo, Characterization of volatile profiles of three colored quinoas based on GC-IMS and PCA, LWT 146 (2021), 111292. https://doi.org/10.1016/j.lwt.2021.111292.

[85] Ruiz J., Kiene F., Belda I., Fracassetti D., Marquina D., Navascués E., Calderón F., Benito A., Rauhut D., Santos A., Benito S. (2019). Effects on varietal aromas during wine making: a review of the impact of varietal aromas on the flavor of wine. Appl. Microbiol. Biotechnol..

[86] Sumby K.M., Grbin P.R., Jiranek V. (2010). Microbial modulation of aromatic esters in wine: current knowledge and future prospects. Food Chem..

[87] Swiegers J.H., Bartowsky E.J., Henschke P.A., Pretorius I.S. (2005). Yeast and bacterial modulation of wine aroma and flavour. Aust. J. Grape Wine Res..

[88] Qian X., Jia F., Cai J., Shi Y., Duan C., Lan Y. (2021). Characterization and evolution of volatile compounds of cabernet sauvignon wines from two different clones during oak barrel aging. Foods.

[89] Coelho E., Domingues L., Teixeira J.A., Oliveira J.M., Tavares T. (2019). Understanding wine sorption by oak wood: modeling of wine uptake and characterization of volatile compounds retention. Food Res. Int..

[90] G. Ramirez Ramirez, S. Lubbers, C. Charpentier, M. Feuillat, A. Voilley, & D. Chassagne, aroma compound sorption by oak wood in a model wine, J. Agric. Food Chem. 49 (2001), 3893–3897. . 10.1021/jf001334a 11513685

[91] Perestrelo R., Silva C., Câmara J.S. (2019). Madeira wine volatile profile. A Platform to Establish Madeira Wine Aroma Descriptors. Molecules.

[92] Rocha S.M., Rodrigues F., Coutinho P., Delgadillo I., Coimbra M.A. (2004). Volatile composition of baga red wine: assessment of the identification of the would-be impact odourants. Anal. Chim. Acta.

[93] Slaghenaufi D., Ugliano M. (2018). Norisoprenoids, sesquiterpenes and terpenoids content of valpolicella wines during aging: investigating aroma potential in relationship to evolution of tobacco and balsamic aroma in aged wine. Front. Chem..

[94] Fernández de Simón B., Cadahía E., del Álamo M., Nevares I. (2010). Effect of size, seasoning and toasting in the volatile compounds in toasted oak wood and in a red wine treated with them. Anal. Chim. Acta.

