# Supplementary material for: Application of toasted vine-shoot chips and ultrasound treatment in the ageing of Primitivo wine
Source: Ultrason Sonochem. 2024 Feb 24;104:106826. doi: 10.1016/j.ultsonch.2024.106826 (PMC10909903; doi:10.1016/j.ultsonch.2024.106826)
Supplement: Supplementary data 1 [file mmc1.docx]

| **Table S1.** Incidence of sampling time (Tm, 7, 14, 21, 28 days) and treatments (Tr) on volatile organic compounds (µg L^-1^) of Primitivo wines. | | | | | | | | | | | | | | | | |
| --- | --- | --- | --- | --- | --- | --- | --- | --- | --- | --- | --- | --- | --- | --- | --- | --- |
| **Compounds** | **C** | | | | | **I** | | | | **U+I** | | | | **Tm** | **Tr** | **Tm*Tr** |
|  | **7** | **14** | **21** | | **28** | **7** | **14** | **21** | **28** | **7** | **14** | **21** | **28** |  |  |  |
| **Alcohols** |  |  |  | |  |  |  |  |  |  |  |  |  |  |  |  |
| Propanol | ^*^18.7±0.1^cd^ | 18.4±2.0^cd^ | 22.7±2.9^bcd^ | | 23.0±4.3^bcd^ | 25.6±0.8^bc^ | 24.6±1.0^bc^ | 24.9± 3.8^bc^ | 16.3±0.8^d^ | 28.5±1.7^b^ | 55.5±3.9^a^ | 22.6±1.0^bcd^ | 21.4±2.4^bcd^ | *** | *** | *** |
| Isobutanol | 320.7±5.95^abcd^ | 294.8±6.7^cde^ | 323.7±5.6^abcd^ | | 323.6±45.9^abcd^ | 326.2±16.4^abcd^ | 338.3±18.8^abc^ | 369.1±8.0^a^ | 243.4±8.2e | 359.0±12.1^ab^ | 290.5±14.5^cde^ | 301.4±8.8^bcde^ | 263.1±47.2^de^ | *** | ns | *** |
| 1-Butanol | 7.0±0.5^abc^ | 7.1±0.6^ab^ | 7.9±0.4^a^ | | 6.7±0.9^abc^ | 7.7±0.4a | 7.0±0.4^abc^ | 7.5±0.3^ab^ | 5.64 ± 0.47c | 8.1±0.3^a^ | 7.6±0.3^a^ | 7.1±0.2^ab^ | 6.1±0.9^bc^ | *** | ns | *** |
| Isoamyl alcohol | 8129.3±69.8^bc^ | 7267.3±159.0^cd^ | 7661.9±167.5^bc^ | | 7696.0±726.7^bc^ | 8861.9±212.9^ab^ | 7947.9±660.5^bc^ | 8401.2±166.0^abc^ | 6142.8±380.4^d^ | 9653.2±498.2^a^ | 7092.3±487.0^cd^ | 7190.8±38.2^cd^ | 6156.1±971.4^d^ | *** | ns | *** |
| 4-Methyl-1-pentanol | 2.6±0.1^c^ | nd | 5.7±0.2^a^ | | 2.2±0.1^de^ | 2.5±0.2^cd^ | nd | 3.4±0.1^b^ | 2.2±0.2^e^ | 1.9±0.1^ef^ | nd | 2.2±0.01^e^ | 1.7±0.16^f^ | *** | *** | *** |
| 3-Methyl-1-pentanol | 6.2±0.2^b^ | 5.3±0.5^bc^ | 6.3±0.2^b^ | | 5.7±0.1^b^ | 6.4±0.1^b^ | 4.0±0.4^d^ | 6.2±0.2^b^ | 4.1±0.3^cd^ | 7.7±0.5^a^ | 5.33±0.8^bc^ | 5.3±0.2^bc^ | 4.2±0.7^cd^ | *** | ** | *** |
| 1-Hexanol | 94.7±4.5^abcd^ | 98.0±2.7^abc^ | 96.3±1.1^abcd^ | | 99.9±8.0^ab^ | 98.1±2.2^abc^ | 96.9±5.2^abc^ | 101.2±2.1^ab^ | 81.3± 5.3d | 108.8±3.5^a^ | 98.0±4.1^abc^ | 87.0±2.1^bcd^ | 84.4±11.4^cd^ | *** | ns | *** |
| 3-Hexen-1-ol | nd | nd | 2.3±0.1^b^ | | nd | 3.2±0.2^a^ | nd | 2.2±0.04^b^ | nd | 2.4±0.1^b^ | nd | 3.2±0.04^a^ | nd | *** | *** | *** |
| 1-Octen-3-ol | 7.5±0.3^bc^ | 7.7±0.4^abc^ | 7.6±0.2^bc^ | | 7.7±0.2^abc^ | 8.3±0.2^ab^ | 7.3±0.2^bcd^ | 8.0±0.02^ab^ | 6.6±0.6^cd^ | 8.2±0.6^ab^ | 7.4±0.03^bc^ | 8.9±0.1^a^ | 6.1± 0.9^d^ | *** | ns | *** |
| Methionol | 5.1±0.2^c^ | 3.4±0.1^f^ | nd | | nd | 6.1±0.9^b^ | nd | nd | 2.1±0.1^g^ | 7.9±0.1^a^ | 4.1±7.8^e^ | nd | 4.6±0.3^d^ | *** | ns | *** |
| Benzyl Alcohol | 6.6±0.3^cd^ | 5.5±0.20^d^ | 7.6±0.2^bc^ | | 7.7±1.0^bc^ | 6.6±0.5^cd^ | 6.9±0.7^bcd^ | 8.3±0.2^ab^ | 6.3±0.2^cd^ | 9.8±0.4^a^ | 6.5±0.2^cd^ | 8.3±0.2^b^ | 5.7±0.7^d^ | *** | ** | *** |
| 2-Phenylethanol | 2414.1±258.7^bcd^ | 2107.1±110.1^cdef^ | 2293.2±35.4^cdef^ | | 2378.1±275.0^bcde^ | 2491.9±118.0^bc^ | 2054.4±175.9^def^ | 2789.8±47.8^ab^ | 1965.4±59.2^ef^ | 2977.8±133.0^a^ | 2338.4±6.2^cdef^ | 2154.4±33.5^cdef^ | 1909.3±143.3^f^ | *** | ns | *** |
| ***Total*** | **11012.6±305.1^bc^** | **9814.7±262.7^cd^** | **10435.2±209.6^bc^** | | **10550.8±1060.1^bc^** | **11844.6±270.1^ab^** | **10487.3±847.2^bc^** | **11722.0±219.5^ab^** | **8476.6±405.4^d^** | **13173.3±452.0^a^** | **9905.7±520.7^cd^** | **9791.1±52.9^cd^** | **8462.8±1176.9^d^** | ******* | **ns** | ******* |
| **Aldehydes** |  |  |  | |  |  |  |  |  |  |  |  |  |  |  |  |
| Acetaldehyde | 1.8±0.1^g^ | 1.3±0.03^g^ | 2.7±0.04^ef^ | | 3.4±0.2^cde^ | 3.7±0.1^cd^ | 2.0±0.1^fg^ | 4.8±0.1^a^ | 3.1±0.1^de^ | 3.7±0.2^cd^ | 2.7±0.1^ef^ | 3.9±0.1^bc^ | 4.5±0.8^ab^ | *** | *** | *** |
| Octanal | 5.0±0.3^bc^ | 2.6±0.1^def^ | 3.8±0.1^cd^ | | 3.3±0.2^de^ | 1.7±0.2^f^ | 9.2±1.1^a^ | 5.3±0.1^b^ | 8.8±0.9^a^ | 3.2±0.1^de^ | 3.8±0.4^cd^ | 3.2±0.04^de^ | 2.1±0.2^ef^ | *** | *** | *** |
| Nonanal | 28.2±2.0^cd^ | 12.4±1.0^de^ | 16.9±0.4^cde^ | | 10.2±1.6^e^ | 32.6±2.2^bc^ | 93.0±6.0^a^ | 28.6±0.6^cd^ | 86.1±17.6^a^ | 46.2±6.7^b^ | 46.4±4.0^b^ | 16.4±0.2^cde^ | 6.9±0.6^e^ | *** | *** | *** |
| Furfural | 3.5±0.2^d^ | 4.6±0.2^d^ | 5.7±0.2^d^ | | 8.3±0.8^d^ | 60.3±3.5^ab^ | 56.9±5.4^abc^ | 56.9±1.6^abc^ | 46.4±4.0^c^ | 67.7±2.9^a^ | 57.1±3.4^abc^ | 53.5±4.6^bc^ | 51.9±1.4^bc^ | *** | *** | *** |
| Decanal | 8.1±0.1^de^ | 5.6±0.7^f^ | 5.6±0.4^f^ | | 11.7±0.6^a^ | 12.4±1.2^ab^ | 11.1±0.6^a^ | 11.7±0.4^abc^ | 6.5±0.2^ef^ | 10.0±0.3^cd^ | 13.3±0.2^a^ | 4.9±0.2^f^ | 5.1±0.9^f^ | *** | *** | *** |
| Benzaldehyde | 7.9±0.2^e^ | 7.7±0.3^e^ | 7.6±0.2^e^ | | 8.5±1.0^de^ | 11.7±0.6^bc^ | 11.5±0.5^bc^ | 14.7± 0.2^a^ | 10.2±0.3^cd^ | 15.0±0.7^a^ | 11.8±0.7^bc^ | 12.7±0.1^b^ | 10.6±1.6^c^ | *** | *** | *** |
| 5-Methylfurfural | nd | nd | nd | | nd | 17.0±0.9^ab^ | 15.5±1.4^ab^ | 18.1±0.6^ab^ | 14.7±1.3^b^ | 19.4±1.1^a^ | 15.6±0.4^ab^ | 17.1±2.0^ab^ | 17.1±0.1^ab^ | *** | *** | ** |
| ***Total*** | **54.5±2.4^f^** | **34.2±1.1^f^** | **42.1±2.1^f^** | | **45.4±2.0^f^** | **139.5±8.7^d^** | **199.2±5.0^a^** | **140.1±3.2^d^** | **175.9±20.0^b^** | **165.1±9.1^bc^** | **150.8±4.09^cd^** | **111.8±6.3^e^** | **98.3±4.8^e^** | ******* | ******* | ******* |
| **Acetate esters** |  |  |  | |  |  |  |  |  |  |  |  |  |  |  |  |
| Methyl acetate | 7.0±0.4^bc^ | 5.9±0.4^c^ | 7.2±0.2^b^ | | 6.7±0.5^bc^ | 7.2±0.3^b^ | 7.3±0.6^b^ | 9.1±0.1^a^ | 7.3±0.4^b^ | 9.7±0.4^a^ | 6.7±0.4^bc^ | 6.4±0.1^bc^ | 6.2±0.8^bc^ | *** | *** | *** |
| Ethyl acetate | 690.8±15.4^a^ | 607.3±12.0^bc^ | 527.3±14.1^de^ | | 594.3±42.8^bcd^ | 648.7±33.8^ab^ | 605.4±10.4^bc^ | 544.3±14.9^cd^ | 464.4±38.0^ef^ | 568.9±38.3^cd^ | 460.1±20.7^ef^ | 379.9±0.3^g^ | 404.9±33.6^fg^ | *** | *** | ** |
| Isobutyl acetate | 5.4±0.03^a^ | 3.5±0.3^bcd^ | 4.1±0.1^b^ | | 3.7±0.05^bc^ | 3.3±0.2^cd^ | 3.6±0.2^bcd^ | 3.5±0.1^bcd^ | 4.1±0.3^b^ | 4.1±0.3^b^ | 3.3±0.4^cd^ | 3.0±0.03^de^ | 2.5±0.1^e^ | *** | *** | *** |
| Isoamyl acetate | 337.8±21.5^a^ | 323.3±5.5^a^ | 145.6±22.7^c^ | | 229.4±6.5^b^ | 246.3±11.5^b^ | 159.7±3.8^c^ | 136.9±4.5^c^ | 214.7±4.0^b^ | 217.8±27.4^b^ | 226.4±30.2^b^ | 139.8±0.81^c^ | 131.6±9.5^c^ | *** | *** | *** |
| Hexyl acetate | 6.7±0.01^a^ | 6.0±0.3^ab^ | 4.1±0.1^bcd^ | | 4.2±0.2^bcd^ | 4.6±0.4^bcd^ | 3.1±0.3^d^ | 3.9±0.1^cd^ | 5.8±0.5^abc^ | 5.7±0.2^abc^ | 5.9±2.0^ab^ | 3.1±0.02^d^ | 5.5±1.0^abc^ | *** | ** | *** |
| Phenethyl acetate | 103.5±2.2^a^ | 92.7±2.2^abc^ | 100.9±1.7^ab^ | | 102.3±6.3^a^ | 95.0±5.0^bc^ | 88.0±8.0^abc^ | 102.0±2.0^a^ | 83.7±6.4^cd^ | 100.8±5.2^ab^ | 84.4±1.9bcd | 90.3±1.8^abc^ | 69.5±10.8^d^ | *** | ** | *** |
| Ethyl isopentyl succinate | 5.8±0.4^ef^ | 5.7±0.3ef | 13.2±0.2^a^ | | 9.3±1.1^bc^ | 7.1±0.3^de^ | 6.6±0.5^ef^ | 10.1±0.4^b^ | 8.2±0.6^cd^ | 7.1±0.1^de^ | 5.5±0.2^f^ | 8.3±0.05^cd^ | 6.1±0.80^ef^ | *** | *** | *** |
| ***Total*** | **1157.1±33.8^a^** | **1044.3±14.6^b^** | **802.5±26.9^e^** | | **949.9±51.4^bcd^** | **1012.3±17.8^bc^** | **873.5±21.8^de^** | **809.8±19.7^e^** | **788.1±46.3^e^** | **914.2±50.6^bcd^** | **792.3±42.6^e^** | **630.7±1.2^f^** | **626.3±44.4^f^** | ******* | ******* | ****** |
| **Ethyl Esters** |  |  |  | |  |  |  |  |  |  |  |  |  |  |  |  |
| Ethyl butyrate | 44.1±3.9^a^ | 42.7±3.1^ab^ | 29.2±5.2^cde^ | | 34.3±2.6^bc^ | 33.8±3.2^c^ | 31.8±3.2^cd^ | 29.4±0.4^cde^ | 30.6±0.9^cde^ | 35.4±1.5^abc^ | 28.2±1.6^cde^ | 21.8±4.3^e^ | 24.6±1.8^de^ | *** | *** | * |
| Ethyl isovalerate | 2.2±0.07^a^ | 2.2±0.1^a^ | 2.0±0.1^ab^ | | 1.9±0.2^abc^ | nd | 1.6±0.2^cd^ | 1.5±0.03^d^ | 1.8±0.1^bcd^ | nd | 1.8±0.20^bcd^ | 1.6±0.01^d^ | 1.6±0.2^cd^ | *** | *** | *** |
| Ethyl hexanoate | 229.2±16.1^bc^ | 294.1±14.6^a^ | 97.1±1.1^de^ | | 173.6±6.5^bc^ | 148.8± 8.3^cd^ | 82.7±5.3^e^ | 63.2±1.5^e^ | 180.7±12.6^bc^ | 165.3±27.7^c^ | 151.8±51.1^cd^ | 66.8±9.3^e^ | 106.3±18.7^de^ | *** | *** | *** |
|  |  |  |  | |  |  |  |  |  |  |  |  |  |  |  |  |
|  |  |  |  | |  |  |  |  |  |  |  |  |  |  |  |  |
| **Table S1.** *Continued* | | | | | | | | | | | | | | | | |
| Ethyl heptanoate | 6.0±0.2^a^ | 5.0±0.3^b^ | 3.3±0.04^e^ | | 4.6±0.5^bc^ | 1.8±0.1^g^ | 3.4±0.4^de^ | 1.7±0.02^g^ | 4.2±0.3^cd^ | 3.1±0.2^e^ | 4.2±0.3^bc^ | 2.92±0.03^ef^ | 2.3± 0.1^fg^ | *** | ns | *** |
| 2-Hydroxyethyl propionate | 43.7±2.4^d^ | 47.7±1.9^cd^ | 68.1±0.8^ab^ | | 75.6±11.8^a^ | 46.7±2.4^cd^ | 56.3±3.4^bcd^ | 67.5±2.1^ab^ | 57.4±1.6^bcd^ | 53.6±2.3^bcd^ | 64.6±4.0^ab^ | 64.2±1.1^ab^ | 59.4±9.6^bc^ | *** | ns | *** |
| Ethyl octanoate | 858.8±40.0^b^ | 1241.2±25.8^a^ | 400.3±53.1^de^ | | 766.2±86.5^bc^ | 252.7±9.6^efg^ | 204.6±15.2^fg^ | 173.4±8.2^g^ | 253.31±34.4^efg^ | 761.7±73.2^bc^ | 817.0±104.5^b^ | 355.0±19.3^def^ | 493.4±64.9^d^ | *** | *** | *** |
| Heptyl formate | 31.4±0.9^bcd^ | 31.2±0.5^bcd^ | 33.0±0.7^abc^ | | 34.6±3.0^ab^ | 32.7±2.6^abc^ | 30.0±1.9^bcd^ | 36.5±0.3^a^ | 27.0±1.8^d^ | 30.8±2.0^bcd^ | 30.3±0.2^bcd^ | 31.6±0.3^abcd^ | 28.2±2.1^cd^ | *** | ** | *** |
| Ethyl 3-hydroxybutyrate | 1.5±0.06^d^ | nd | 2.3±0.05^a^ | | nd | 2.1±0.1^b^ | nd | 1.9±0.02^c^ | nd | 2.0±0.1^bc^ | nd | 2.0±0.1^bc^ | nd | *** | ns | *** |
| Ethyl nonanoate | 7.5±0.2^cde^ | 6.4±0.2^ef^ | 7.3±0.1^cdef^ | | 10.9±0.3^a^ | 7.2±0.2^def^ | 7.7±0.4^cde^ | 5.7± 0.1^g^ | 9.8±0.7^ab^ | 9.1±0.4^bc^ | 8.9±1.2^bcd^ | 7.6±0.1^cde^ | 9.0±1.7^bcd^ | *** | ** | *** |
| Ethyl decanoate | 268.8±9.4^a^ | 142.6±2.2^c^ | 162.2±14.7^c^ | | 237.9±25.1^b^ | 56.7±3.3^ef^ | 43.4±5.6^f^ | 42.2±2.8^f^ | 76.2±6.6^de^ | 88.6±6.2^d^ | 64.6±8.9^def^ | 53.6±6.8^ef^ | 58.1±7.0^ef^ | *** | *** | *** |
| Diethyl succinate | 308.8±18.40^e^ | 336.5±6.8^de^ | 411.0±13.9^abc^ | | 423.3±33.7^ab^ | 334.4±21.3^de^ | 358.0±38.7^bcde^ | 441.8±1.0^abc^ | 347.9±11.1^cde^ | 373.0±2.6^bcde^ | 352.3±0.2^cde^ | 395.5±10.4^abcd^ | 338.4±42.1^de^ | *** | ns | *** |
| ***Total*** | **1802.0±51.8^b^** | **2149.6±46.4^a^** | **1216.0±53.1^ef^** | | **1763.0±154.3^bc^** | **916.9±19.5^gh^** | **819.6±60.7^h^** | **864.8±10.6^h^** | **988.9±40.4^gh^** | **1522.7±102.3^cd^** | **1524.0±157.7^cd^** | **1002.7±26.8^fgh^** | **1121.3±102.3^fg^** | ******* | ******* | ******* |
| **Other esters** |  |  |  | |  |  |  |  |  |  |  |  |  |  |  |  |
| Methyl 2-furoate | nd | nd | nd | | nd | 5.83 ± 0.12^bcd^ | 5.7±0.5^cd^ | 6.5±0.3^abc^ | 5.0±0.4^de^ | 6.9±0.5^a^ | 4.43±7.9^e^ | 6.6±0.1^ab^ | 6.2±0.2^abc^ | *** | *** | *** |
| Ethyl 2-furoate | nd | nd | nd | | nd | 4.1±0.2^a^ | 3.1±0.7^bc^ | 4.2±0.1^a^ | 2.6±0.1^c^ | 3.9±0.3^ab^ | 2.7±0.2^c^ | 3.1±0.04^bc^ | 2.6±0.4^c^ | *** | *** | *** |
| ***Total*** | **nd** | **nd** | **nd** | | **nd** | **10.0±0.3^ab^** | **8.8±1.1^bc^** | **10.7±0.3^a^** | **7.6±0.4^cd^** | **10.7±0.8^a^** | **7.2±0.5^d^** | **9.8±0.1^ab^** | **8.8±0.6^bc^** | ******* | ******* | ******* |
| **Ketones** |  |  |  | |  |  |  |  |  |  |  |  |  |  |  |  |
| 2-Octanone | 3.5±0.2^ab^ | 2.4±0.2^ef^ | 2.9±0.1^bcde^ | | 3.4±0.4^abc^ | 2.7±0.05^de^ | 3.3±0.1^abcd^ | 2.9±0.1bcde | 3.72 ± 0.27a | 2.6±0.1^e^ | 3.3±0.4^abcd^ | 2.8±0.02^cde^ | 1.9±0.3^f^ | ns | *** | *** |
| Acetoin | 5.20±0.3^d^ | 3.3±0.3^e^ | 6.8±0.1^b^ | | 8.6±0.9^a^ | 3.8±0.2^e^ | 6.2±0.4^bcd^ | 5.5±0.1^cd^ | 6.6±0.4^bc^ | 3.1±0.2^e^ | 5.5±0.2^d^ | 6.9±0.1^b^ | 5.6±0.6^cd^ | *** | *** | *** |
| ***Total*** | **8.7±0.3^cd^** | **5.8±0.5^f^** | **9.7±0.1^bc^** | | **12.0±1.3^a^** | **6.5±0.2^ef^** | **9.4±0.4^bc^** | **8.4±0.2^cd^** | **10.4±0.7^b^** | **5.7±0.3^f^** | **8.8±0.3^bcd^** | **9.7±0.1^bc^** | **7.4±0.8^de^** | ******* | ******* | ******* |
| **Terpenes** |  |  |  | |  |  |  |  |  |  |  |  |  |  |  |  |
| Linalool | 14.5±0.1^abc^ | 15.6±0.3^abc^ | 14.9±0.6^abc^ |  | 15.6±1.4^abc^ | 15.3± 0.7^abc^ | 15.0±2.3^abc^ | 17.6±2.4^ab^ | 12.5±1.0^c^ | 17.9±2.5^a^ | 15.3±1.2^abc^ | 17.2±1.3^ab^ | 13.0±2.2^bc^ | ** | ns | * |
| α-Terpineol | nd | nd | nd | | nd | 4.2±0.2^b^ | 2.9±0.2^d^ | 3.7±0.1^bc^ | 3.2±0.1c^d^ | 4.1±0.1^b^ | 3.0±0.2^d^ | 5.3±0.1^a^ | 2.7±0.1^d^ | *** | *** | *** |
| ***Total*** | **14.5±0.1^c^** | **15.6±0.3^bc^** | **14.9±0.6^bc^** | | **15.6±1.4^bc^** | **19.5± 0.7^abc^** | **17.9±2.3^abc^** | **21.3±2.4^ab^** | **15.7±1.0^bc^** | **22.0±2.5^a^** | **18.4±1.3^abc^** | **22.6±1.3^a^** | **15.7±2.2^bc^** | ****** | **ns** | ***** |
| **Noroisoprenoids** |  |  |  | |  |  |  |  |  |  |  |  |  |  |  |  |
| β-Damascenone | 6.9±0.1^ab^ | 6.0±0.9^abc^ | 7.4±0.3^a^ | | 5.1±0.2^c^ | 4.3±0.05^c^ | 5.1±0.4^c^ | 5.3±0.7^bc^ | 5.1±0.2^c^ | 5.7±0.2^bc^ | 4.4±0.4^c^ | 4.8±0.6^c^ | 4.5±1.3^c^ | ** | *** | ** |
| **Carboxylic Acids** |  |  |  | |  |  |  |  |  |  |  |  |  |  |  |  |
| Acetic acid | 55.3±5.9^de^ | 59.9±5.2^cde^ | 77.0±0.2^bc^ | | 102.1±11.2^a^ | 53.3±0.7^e^ | 71.4±9.1^bcde^ | 57.9±0.8^cde^ | 62.8±4.7^cde^ | 87.7±8.3^ab^ | 55.8±1.1^de^ | 74.6±1.3^bcd^ | 63.1±14.8^cde^ | ** | *** | *** |
| Hexanoic acid | 21.8±2.4^b^ | 26.4±2.3^ab^ | 27.9±0.5^ab^ | | 31.7±4.6^a^ | 27.0±1.7^ab^ | 26.7±1.8^ab^ | 30.6±0.5^a^ | 28.2±0.6^ab^ | 30.3±2.8^a^ | 26.5±0.6^ab^ | 30.1±0.4^a^ | 27.8±3.2^ab^ | ** | ns | ** |
| Octanoic acid | 63.2±4.0^cd^ | 74.20±5.9^bc^ | 87.0±1.6^ab^ | | 102.5±16.1^a^ | 61.7±7.4^cd^ | 72.4±7.7^bc^ | 87.6±3.7^ab^ | 71.1±4.8^bcd^ | 65.9±1.4^cd^ | 52.2±1.0^d^ | 64.3±0.8^cd^ | 51.9 ± 5.0^d^ | *** | *** | *** |
| Nonanoic acid | 4.9±0.20^c^ | nd | 6.5±0.20^b^ | | nd | nd | 12.0±1.8^a^ | nd | 7.3±0.7^b^ | 6.3±0.3^b^ | nd | 7.1±0.1^b^ | nd | *** | *** | *** |
| n-Decanoic acid | 12.1±0.33^cd^ | 15.1±0.8^cd^ | 26.5±3.5^a^ | | 21.6±3.1^b^ | 4.9±0.3^e^ | 13.2±2.2^cd^ | 23.7±0.5^ab^ | 22.3±1.2^ab^ | 10.6±0.6^d^ | 13.2±0.9^cd^ | 16.4±0.3^c^ | 5.4 ± 0.1^e^ | *** | *** | *** |
| ***Total*** | **157.4±11.9^de^** | **175.6±10.3^cde^** | **225.0±2.3^ab^** | | **257.9±34.9^a^** | **147.0±10.0^e^** | **195.8±18.4^bcd^** | **199.9±5.3^bcd^** | **191.7±3.0^bcd^** | **200.9±6.4^bc^** | **147.7±6.3^e^** | **192.4±2.7^bcd^** | **148.1±23.0^e^** | ******* | ******* | ******* |
| ***TOTAL*** | **14213.8±344.3^ab^** | **13245.7±324.1^bc^** | **12752.7±176.0^bc^** | | **13599.7±1257.5^bc^** | **14100.6±304.9^ab^** | **12616.8±935.5^bcd^** | **13782.4±249.4^bc^** | **10659.6±499.0^de^** | **16020.6±528.7^a^** | **12559.9±720.0^bcd^** | **11775.9±38.1^cde^** | **10493.3±1303.4^e^** | ******* | ***** | ******* |

^*^In row, data followed by different letters indicate statistically significant differences at *p <* 0.05 according to two-way ANOVA with interaction (Tm*Tr) followed by Tukey’s test. Significance: ns, *, **, and ***, not significant or significant at *p <* 0.05, *p <* 0.01, or *p <* 0.001, respectively. Average value ± standard deviation (n = 3). *Abbreviations*: C, control wine; I, wine with infusion of vine-shoot chips; U + I, wine with ultrasound and infusion of vine-shoot chips; nd, not detected.

**Table S2.**  OAVs of aroma compounds from Primitivo wines.

| Compounds | Odor threshold (μg/L) | Odor description | C | | | | I | | | | U+I | | | | | | |
| --- | --- | --- | --- | --- | --- | --- | --- | --- | --- | --- | --- | --- | --- | --- | --- | --- | --- |
|  |  |  | **7** | **14** | **21** | **28** | **7** | **14** | **21** | **28** | **7** | **14** | **21** | **28** |  | |  |
| Octanal | 0.7 ^[1]^ | Green, fat ^[10]^ | 7.0 | 3.8 | 5.4 | 4.7 | 2.5 | 13.1 | 7.6 | 12.5 | 4.6 | 5.5 | 4.5 | 3.0 |  |  |  |
| Nonanal | 1.1 ^[2]^ | Floral, green, lemon-like ^[11]^ | 25.7 | 11.3 | 15.3 | 9.2 | 29.6 | 84.6 | 26.0 | 78.2 | 42.0 | 42.2 | 14.9 | 6.3 |  |  |  |
| Decanal | 2 ^[3]^ | Floral, soap ^[12]^ | 4.1 | 2.8 | 2.7 | 5.9 | 6.2 | 5.5 | 5.8 | 3.3 | 5.0 | 6.7 | 2.5 | 2.6 |  |  |  |
| 1-Octen-3-ol | 1 ^[4]^ | Mushroom ^[4]^ | 7.5 | 7.7 | 7.6 | 7.7 | 8.3 | 7.3 | 8.0 | 6.6 | 8.2 | 7.4 | 8.9 | 6.1 |  |  |  |
| 2-Phenylethanol | 1100 ^[5]^ | Floral, rose, honey ^[4]^ | 2.2 | 1.9 | 2.1 | 2.2 | 2.3 | 1.9 | 2.5 | 1.8 | 2.7 | 2.1 | 2.0 | 1.7 |  |  |  |
| Isoamyl acetate | 26.2 ^[6]^ | Banana ^[6]^ | 12.9 | 12.3 | 5.6 | 8.8 | 9.4 | 6.1 | 5.2 | 8.2 | 8.3 | 8.6 | 5.3 | 5.0 |  |  |  |
| Ethyl butyrate | 18 ^[6]^ | Apple ^[6]^ | 2.5 | 2.4 | 1.6 | 1.9 | 1.9 | 1.8 | 1.6 | 1.7 | 2.0 | 1.6 | 1.2 | 1.4 |  |  |  |
| Ethyl isovalerate | 1.55 ^[6]^ | Fruity ^[6]^ | 1.4 | 1.4 | 1.3 | 1.2 | – | 1.1 | 1.0 | 1.2 | – | 1.2 | 1.0 | 1.1 |  |  |  |
| Ethyl hexanoate | 4 ^[6]^ | Apple peel fruit ^[6]^ | 57.3 | 73.5 | 24.3 | 43.4 | 37.2 | 20.7 | 15.8 | 45.2 | 41.3 | 37.9 | 16.7 | 26.6 |  |  |  |
| Ethyl heptanoate | 2 ^[7]^ | Fruity and apple ^[13]^ | 2.7 | 2.3 | 1.5 | 2.1 | 0.8 | 1.6 | 0.8 | 1.9 | 1.4 | 1.9 | 1.3 | 1.0 |  |  |  |
| Ethyl octanoate | 1.6 ^[6]^ | Fruity, fat ^[6]^ | 536.8 | 775.7 | 250.2 | 478.9 | 157.9 | 127.9 | 108.4 | 408.3 | 476.1 | 510.7 | 221.9 | 308.4 |  |  |  |
| Linalool | 6 ^[8]^ | Citrus, floral, sweet,  grape-like ^[4]^ | 2.4 | 2.6 | 2.5 | 2.6 | 2.5 | 2.5 | 2.9 | 2.1 | 3.0 | 2.6 | 2.9 | 2.2 |  |  |  |
| β-Damascenone | 0.05 ^[9]^ | Sweet, fruit ^[14]^ | 138.2 | 119.3 | 147.9 | 102.0 | 85.8 | 103.0 | 107.0 | 102.0 | 113.8 | 88.4 | 96.6 | 89.8 |  |  |  |

– OAV < 1. *Abbreviations*: C, control wine; I, wine with infusion of vine-shoot chips; U + I, wine with ultrasound and infusion of vine-shoot chips. References: Odor threshold were determined in water: ^[1]^ Pino & Mesa, 2006, ^[2]^ Wen et al., 2014, ^[3]^ Fan, Xu, Jiang, Li, 2010, ^[4]^ Wu et al., 2016, ^[5]^ Buttery, Turnbaugh, Ling, 1988, ^[7]^ Takeoka, Flath, Mon, Teranishi, Guentert, 1990, ^[8]^ Buttery, Teranishi, Ling, Turnbaugh, 1990; odor threshold were determined in 9% ethanol/water solution: ^[6]^ Niu et al., 2019; odor threshold were determined 10% ethanol/water solution: ^[9]^ Guth, 1997; ^[10]^ Song et al., 2021; ^[11]^ Guo et al., 2021; ^[12]^ de-la-Fuente-Blanco, Ferreira, 2020; ^[13]^ Fan et al., 2010; ^[14]^ Sánchez-Palomo, Delgado, Ferrer, Viñas, 2019.

**References**

1. J.A. Pino, J. Mesa. Contribution of volatile compounds to mango (*Mangifera indica* L.) aroma. Flavour Frag. J. 21 (2006), 207–213. <https://doi.org/10.1002/ffj.1703>
2. Y.Q. Wen, F. He, B.Q. Zhu, Y.B. Lan, Q.H. Pan, C.Y. Li, M.J. Reeves, J. Wang, Free and glycosidically bound aroma compounds in cherry (*Prunus avium* L.), Food Chem. 152 (2014), 29–36. <https://doi.org/10.1016/j.foodchem.2013.11.092>
3. W. Fan, Y. Xu, W. Jiang, J. Li, Identification and quantification of impact aroma compounds in 4 nonfloral Vitis vinifera varieties grapes, J. Food Sci. 75 (2010), S81–S88. <https://doi.org/10.1111/j.1750-3841.2009.01436.x>
4. Y. Wu, S. Duan, L. Zhao, Z. Gao, M. Luo, S. Song, Wenping Xu, C. Zhang, C. Ma, S. Wang, Aroma characterization based on aromatic series analysis in table grapes, Sci. Rep. 6 (2016), 31116. <https://doi.org/10.1038/srep31116>
5. R.G. Buttery, J.G. Turnbaugh, L.C. Ling, Contribution of volatiles to rice aroma, Agric. Food Chem. 36 (1988), 1006–1009. <https://doi.org/10.1021/jf00083a025>
6. Y. Niu, P. Wang, Z. Xiao, J. Zhu, X. Sun, R.Wang, Evaluation of the perceptual interaction among ester aroma compounds in cherry wines by GC–MS, GC–O, odor threshold and sensory analysis: An insight at the molecular level, Food Chem. 275 (2019), 143–153. <https://doi.org/10.1016/j.foodchem.2018.09.102>
7. G.R. Takeoka, R.A. Flath, T.R. Mon, R. Teranishi, M. Guentert, Volatile constituents of apricot (*Prunus armeniaca*), J. Agric. Food Chem. 38 (1990), 471–477. https://doi.org/10.1021/jf00092a031
8. R.G. Buttery, R. Teranishi, L. Ling, C., J.G.Turnbaugh, quantitative and sensory studies on tomato paste volatiles, J. Agric. Food Chem. 38 (1990), 336–340. <https://doi.org/10.1021/jf00091a074>
9. H. Guth. Quantitation and sensory studies of character impact odorants of different white wine varieties, J. Agric. Food Chem. 45 (1997), 3027–3032. <https://doi.org/10.1021/jf970280a>
10. J. Song, Y. Shao, Y. Yan, X. Li, J. Peng, L. Guo, Characterization of volatile profiles of three colored quinoas based on GC-IMS and PCA. LWT 146 (2021), 111292. <https://doi.org/10.1016/j.lwt.2021.111292>
11. X. Guo, C.T. Ho, X. Wan, H. Zhu, Q. Liu, Z. Wen,. Changes of volatile compounds and odor profiles in Wuyi rock tea during processing, Food Chem., 341 (2021), 128230. https://doi.org/10.1016/j.foodchem.2020.128230
12. A. de-la-Fuente-Blanco, V. Ferreira, Gas chromatography olfactometry (GC-O) for the (semi) quantitative screening of wine aroma, Foods 9 (2020), 1892. <https://doi.org/10.3390/foods9121892>
13. W. Fan, Y. Xu, W. Jiang, J. Li, Identification and quantification of impact aroma compounds in 4 nonfloral Vitis vinifera varieties grapes, J. Food Sci. 75 (2010), S81–S88. <https://doi.org/10.1111/j.1750-3841.2009.01436.x>
14. E. Sánchez-Palomo, J.A. Delgado, M.A. Ferrer, M.A.G. Viñas, The aroma of La Mancha Chelva wines: Chemical and sensory characterization, Food Res. Int. 119 (2019), 135–142. https://doi.org/10.1016/j.foodres.2019.01.049


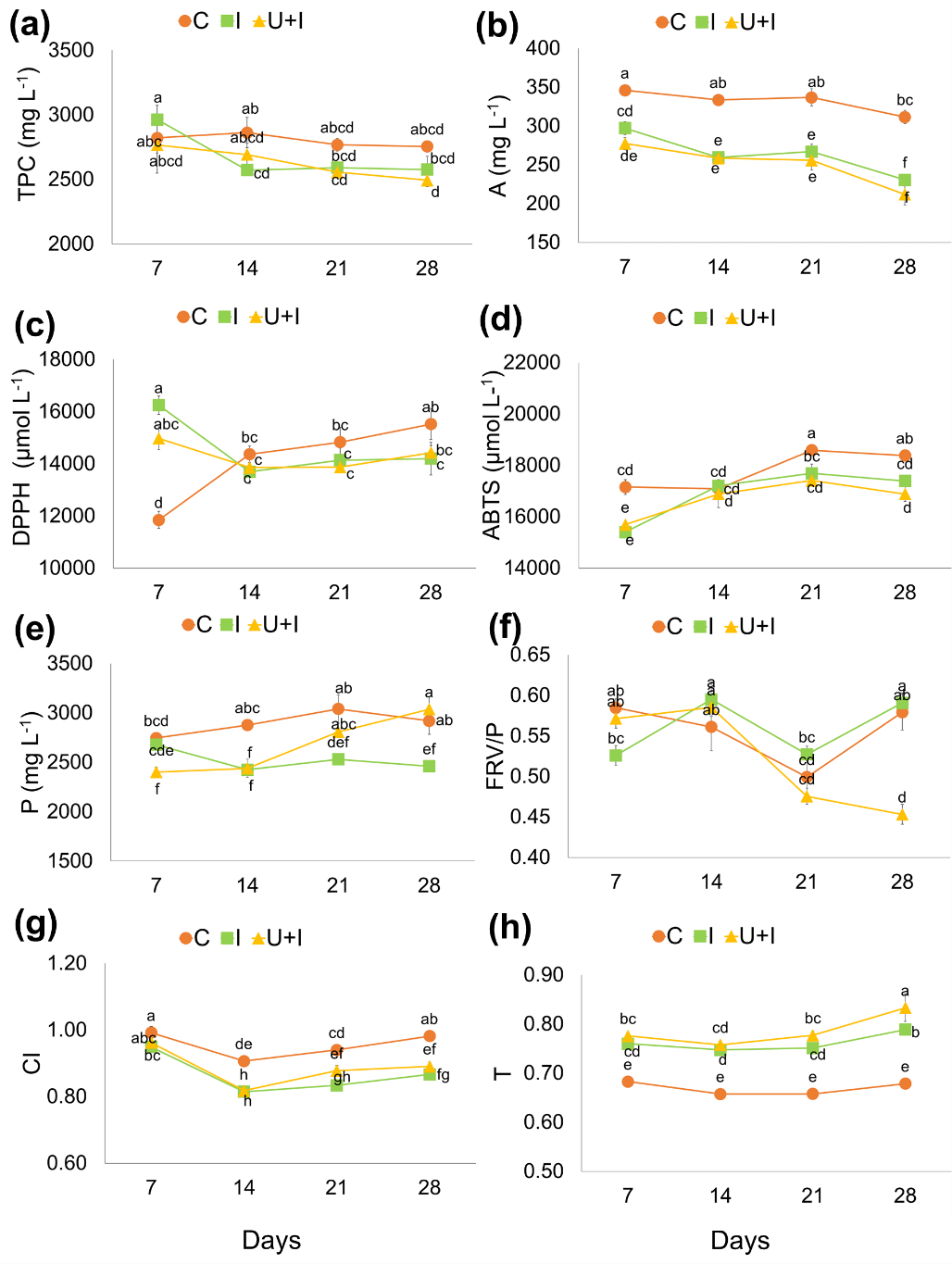


**Figure S1.** Evolution of phenolic parameters in the C, I and U + I wines. (a) total phenolic content (TPC as mg L^-1^ of gallic acid equivalents); (b) anthocyanins (A, as mg L^-1^ of malvidin- 3-glucoside); antioxidant activity by DPPH (c) and ABTS (d) assay; (e) proanthocyanidins (P, as mg L^-^ ^1^ of cyanidin chloride); (f) ratio of flavans reactive with vanillin and proanthocyanidins (FRV/P); (g) colour intensity (CI); (h) tonality. Different letters indicate statistically significant differences at *p* *<* 0.05 according to Tukey’s test.
